# Modelling strategies for the covalent functionalization of 2D phosphorene†
†Electronic supplementary information (ESI) available. See DOI: 10.1039/c8dt03628d


**DOI:** 10.1039/c8dt03628d

**Published:** 2018-11-12

**Authors:** Andrea Ienco, Gabriele Manca, Maurizio Peruzzini, Carlo Mealli

**Affiliations:** a Istituto di Chimica dei Composti Organometallici – Consiglio Nazionale delle Ricerche (CNR-ICCOM) , Via Madonna del Piano 10 , 50019 , Sesto Fiorentino (FI) , Italy . Email: andrea.ienco@iccom.cnr.it ; Email: gabriele.manca@iccom.cnr.it

## Abstract

This paper is a comparative outline of the potential acid–base adducts formed by an unsaturated main group or transition metal species and P atoms of phosphorene (P_*n*_), which derives from black phosphorus exfoliation. Various possibilities of attaining a realistic covalent functionalization of the 2D material have been examined *via* DFT solid state calculations. The distribution of neighbor P atoms at one side of the sheet and the reciprocal directionalities of their lone pairs must be clearly understood to foreshadow the best possible acceptor reactants. Amongst the latter, the main group BH_3_ or I_2_ species have been examined for their intrinsic acidity, which favors the periodic mono-hapto anchoring at P_*n*_ atoms. The corresponding adducts are systematically compared with other molecular P donors from a phosphine to white phosphorus, P_4_. Significant variations emerge from the comparison of the band gaps in the adducts and the naked phosphorene with a possible electronic interpretation being offered. Then, the P_*n*_ covalent functionalization has been analyzed in relation to unsaturated metal fragments, which, by carrying one, two or three vacant σ hybrids, may interact with a different number of adjacent P atoms. For the modelling, the concept of *isolobal analogy* is important for predicting the possible sets of external coligands at the metal, which may allow the anchoring at phosphorene with a variety of hapticities. Structural, electronic, spectroscopic and energy parameters underline the most relevant pros and cons of some new products at the 2D framework, which have never been experimentally characterized but appear to be reasonably stable.

## Abstract 2

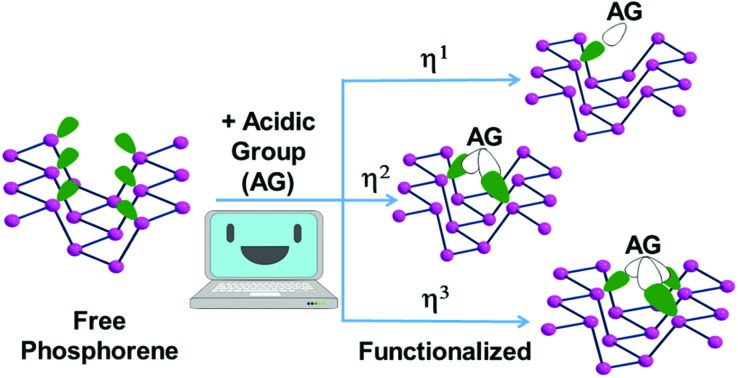
This paper is a comparative outline of the potential acid–base adducts formed by an unsaturated main group or transition metal species and P atoms of phosphorene (P_*n*_), which derives from black phosphorus exfoliation.

## Introduction

Until a few years ago, graphene was unique in the field of 2D materials for its electronic properties stemming from the extended π-delocalization. The consequent electrical and thermal conductivity, in association with the low density and high flexibility of the sheets, predisposes graphene for a plethora of innovative technological applications.^1^ Other 2D materials have been recently investigated, examples being transition metal dicalchogenides^2^ or elemental species such as silicene, germanene^3^ and phosphorene (P_*n*_).^4^ The lone pair at each atom of the latter species is apparently inconsistent with a major electron delocalization similar to that of graphene, thus justifying experimental band gaps up to 2.0 eV.^5^ The gap may be tuned by introducing perturbative effects, a first example being the progressive stacking of the sheets in an ideal reconstruction of the parent black phosphorus material, whose band gap is only 0.33 eV.^6^ Incidentally, the latter value is attainable by combining only ten P_*n*_ sheets.^4^ In any case, in view of the lack of any realistic covalent bond between the layers, the assembly is mainly attributed to the dispersion forces, which also overcome the electron repulsions between the sets of P lone pairs at any two facing sheets. The prevailing attraction is substantiated by the external forces which must be applied to exfoliate black phosphorus, examples being those of micro-mechanical nature, laser irradiation,^4^,^7^ or sonication in solution.^8^ Importantly, the tunable band gap for a limited number of stacked layers can be the basis of new useful electronic devices,^9^ the drawback being the high sensitivity of the material toward atmospheric oxygen and moisture.^10^ For this, protection strategies are generally needed, such as the encapsulation of the material in nanocomposites,^11^ but also covering of the P_*n*_ surface with acidic chemical groups, including transition metal acceptor fragments. In particular, the anchoring of the latter to the surface may also confer relevant catalytic properties on the system, especially if the metal is not yet completely saturated.

In this paper, we will present systematic analyses, first addressing the chemical and structural aspects of the phosphorene P_*n*_ surface and, in particular, the distribution of the P lone pairs, which will interact with σ vacant orbitals at the acidic moieties to be added. The relevance of the donor power at P atoms has already been amply discussed by us relative to the I_3_P formation from the P_4_ + I_2_ reactants.^12^ It emerged that with respect to generic PX_3_ phosphines, the white phosphorus allotrope is definitely a much weaker donor; hence, at this point it becomes important to establish where the phosphorene's lone pairs are positioned compared to the previous limiting cases.

**Fig. 1:**
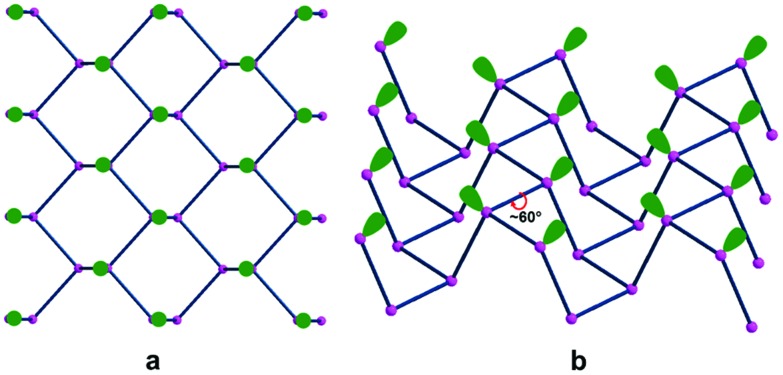
Different views of the phosphorene lone pairs at one side of a 2D material. The top (a) and lateral (b) pictures clearly show how the ragged surface consists of ditches in between opposite zigzag chains with 60° torsions of the two lone pairs associated with a P–P bond.

Fig. 1(a and b) starts illustrating the distribution of the P_*n*_ lone pairs on one side of the 2D material from two viewpoints.

The top view *a* clearly shows how all the lone pairs point out from a P_*n*_ plane although biased by about 60° away from orthogonality, while the lateral view *b* indicates how the two emerging from any P–P bond are reciprocally skewed also by about 60°. Importantly, the rugged P_*n*_ surface consists of parallel zigzag chains with the lone pairs projecting inside the ditches. In principle, any of them may overlap with a mono-functional σ acceptor and, in the case of a metal, contribute to a 2e^–^ donation. The interactions with a multi-functional σ acceptor metal are instead more complex, in view of the simultaneous involvement of two or three neighbor lone pairs, which do not naturally converge into the same point. Still, multiple P–M bonds with one metal seem possible, although they cannot be necessarily equivalent for overlap reasons. Indeed, some geometric adaptation is necessary to allow the multiple-hapticity of phosphorene to a metal.

The mentioned stereochemical aspects will be important in our study of phosphorene's covalent functionalization, which is still substantially unexplored. In fact, many reports have concerned the van der Waals (vdW) functionalization of the P_*n*_ surface, examples being the interaction with either 7,7,8,8-tetra-cyano-*p*-quindodimethane (TCNQ) or a perylene diimide,^13^ which are characterized by charge transfer from the exfoliated black phosphorus to the organic moiety. Other experimental studies were related to the non-covalent adsorption of small molecules (*e.g.*, CO) on P_*n*_, with insufficient stereochemical details about the interactions.^14^ One of the few studies of P_*n*_ covalent functionalization with metals has concerned the adduct of the TiL_4_ tetrahedral fragment (L = sulphonic ester), whose stereochemistry has not been illustrated in detail. In fact, characterization of the product was performed by liquid NMR-techniques and the presence of a direct P–Ti bond assessed by Raman spectroscopy.^15^ In other cases, the absorption of some naked metal atoms on the surface has been computationally addressed with a focus on the number of coordinated P atoms and the effects on the band structures.^16^ Solid state calculations are instead reported to corroborate the absorption of a CrO_3_ unit on phosphorene with a unique P–Cr bond completing the tetrahedral coordination of the metal. The linkage of 2.45 Å is relatively large although it is still consistent with covalency, which is further corroborated by the –2.17 eV stabilization energy of the product. No comparison is proposed with any known molecular models of the metal fragment in question.^17^

A detailed experimental and *in silico* study of the P_*n*_ covalent functionalization concerns the micro-mechanically exfoliated black phosphorus over a Si/SiO_2_ substrate. By reaction with an aryl diazonium salt, which releases N_2_, formation of P–aryl covalent bonds is described. In our view, there are still open questions concerning the actual electronic nature of the system. For instance, the P–C bonds imply the formation of local phosphonium cations with a predictably associated anion, whose presence has not been addressed. Remarkably, an uncharged system without any counterion must imply unpaired spins, possibly dispersed throughout the Si/SiO_2_ substrate. In any case, none of the previous points have been focused on at either computational or experimental level.^18^

Based on the summarized state-of-the-art, the covalent functionalization of phosphorene is still bleary, especially from the experimental viewpoint. For this, we propose in this paper a series of potential reactants with suitable electronic features to be absorbed at the P_*n*_ surface through interactions with selected P lone pairs distributed as in Fig. 1. The detailed stereochemical, electronic and energy features of the products appear to be sound for both main group and transition metal reactants, all characterized by residual acceptor capabilities. Initially, our computational approach was based on limited portions of the rugged phosphorene's surface featuring terminal H atoms at the boundaries. However, it became rapidly evident that a model such as P_38_H_16_ could easily undergo geometric distortions that are impossible for an actually periodic P_*n*_ surface; hence actual solid state calculations were performed using the CRYSTAL17 software package.^19^ The increased reliability of the results was verified from structural, spectroscopic and energy viewpoints but also confirmed by the basic electronic properties grasped from the more qualitative molecular modelling.^20^ In summary, the two approaches ensured a continuity of interpretation, which did not only shed light on the covalent bonding enabled at the P_*n*_ surface but also afforded some predictability, which experimental chemists may be willing to try later.

## Results and discussion

### General considerations on the band structure of phosphorene and its affecting parameters

**Fig. 2:**
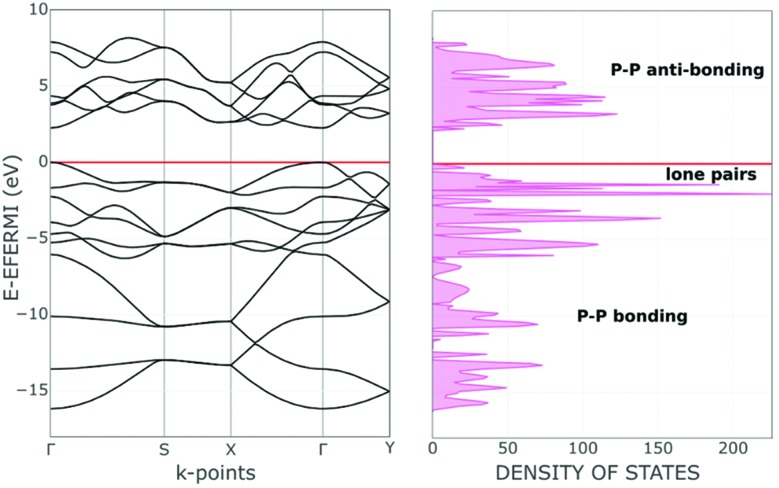
Band and DOS structures of one slab of phosphorene in the valence region.

For a better understanding of phosphorene's electronic properties and their role in the chemical functionalization, some relevant information may stem from the band structure and the density of states (DOS) shown in Fig. 2. The diagrams for the naked 2D material are subdivided into three distinct regions, namely the lower P–P σ bonds, the intermediate frontier P lone pairs and finally the higher and vacant P–P σ* levels. Possible effects on this order are due to structural perturbations^4^,^6^ such as the progressive stacking of P_*n*_ sheets in the reconstruction of the bulk black phosphorus. Also, the basic electronic nature will be modified by some acidic molecules or fragments, which may covalently bind with some P lone pairs at the surface. In any case, the original phosphorene band gap changes due to the possible structural deformations as well as the redistribution of the electron density throughout the surface.

The P_*n*_ slab was fixed by assuming a unit cell of four P atoms or sixteen valence orbitals. Accordingly, the overall band structure at the left side of Fig. 2 combines sixteen different bands, ten of which are populated by the twenty electrons per cell. In the lowest region, six bands stem from the P–P σ bonds, while the next higher four correspond to the lone pairs of the pyramidal P atoms. Although their orientations exclude convergence, the short contacts across the surface determine reciprocal repulsions, which cause a widening of the frontier band >1 eV, as estimated by the B3LYP functional.^21^ At the same time, a 2.26 eV gap is estimated between the valence and conduction regions, with the latter consisting of six bands having the prevailing P–P σ* character. As mentioned, it is sufficient to pile up only ten P_*n*_ sheets to approach the band gap proper of the bulk black phosphorus. The steep variation on stacking is likely a consequence of the cumulative bifacial repulsions between lone pairs with consequent widening of the frontier bands.

**Fig. 3:**
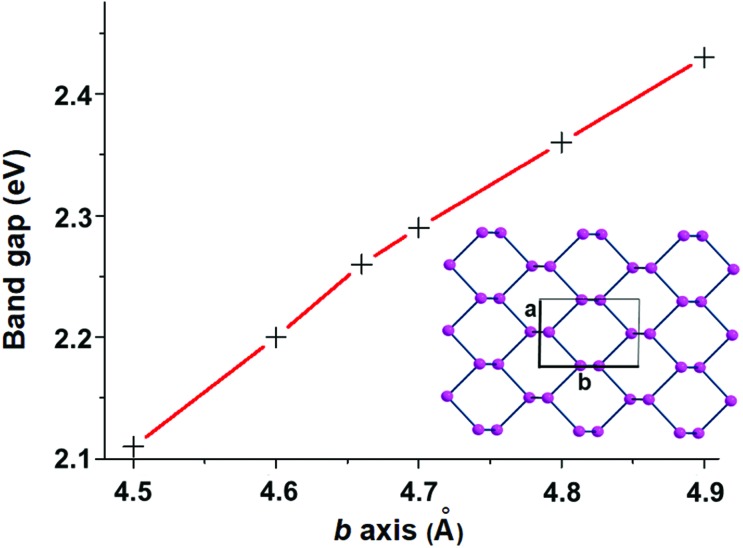
The almost linear relationship between the band gap and the cell parameter *b*.

The covalent functionalization of phosphorene has also electronic and structural consequences on the band structure. For instance, some added reactants may have bands, which fall in between the P_*n*_ valence and conduction ones, an example being the case of the d orbital set of an anchored metal atom. In addition, acidic groups at the surface may withdraw electron density from the P lone pairs with a consequent decrease of their original repulsion effects. As another aspect, groups bound only at one side of a channel, but projecting over it, may become repulsive toward some opposite P atoms inducing a local physical broadening of the channel itself. Obviously, the more frequent is the repetition of the group along the same zigzag chain, the more constant is the widening of the channel itself. To evaluate some possible electronic consequences of these structural effects, we started from the optimized structure of the naked 2D phosphorene with the cell parameter *b* (see the inset of Fig. 3), which provides an indirect evaluation of the channel width. Then, a systematic stretching of *b* (in the range 4.5–4.9 Å) determined the band gap increase from 2.11 to 2.43 eV. The opposite decrease of the band gap occurs upon the stacking of the sheets, as a strategy to reconstruct black phosphorus. This point will be later important for discussing key aspects of phosphorene's functionalization.

### Non-metal Lewis acids in phosphorene's functionalization

One of the most promising mono-functional acidic molecules for σ interaction with the P_*n*_ lone pair can be an uncharged borane, also because it excludes the introduction of any charged group at the surface. Boranes are amply reported in the literature to have affinity for phosphorus donors such as phosphanes,^22^ and may be also apt for interactions with phosphorene. For our *in silico* modelling, the BH_3_ molecule was first chosen to probe its covalent adsorption on P_*n*_; hence various solid state adducts of general formula P_*n*_·*x*(BH_3_) were optimized, with *x* referring to different borane densities at the surface (see below). In a second stage, other acidic molecules or molecular fragments were taken into consideration, being aware that in some cases a heterolytic splitting of the acidic molecule may induce a local separation of charges. For instance, this could be the case of I_2_, whose reaction with a strong R_3_P phosphine donor leads immediately to the ion pair [R_3_PI]^+^[I]^–^,^23^ whereas the white phosphorus allotrope P_4_ has very different consequences such as the cleavage of all six P–P bonds and the formation of the PI_3_ product after a plethora of intermediates.^12^ Also for these reasons, we decided to investigate the behavior of I_2_ at phosphorene to determine which of the alternative adducts is more likely.

The following discussion will be based on computed parameters, the first ones of geometric character. Thus, the strength of the donor–acceptor σ interactions in functionalized P_*n*_ adducts will be evaluated, for instance, from the length of the newly formed bonds and also other more or less evident structural variations occurring at either P_*n*_ or the added acidic molecule. The other important parameter is the estimated binding energy BE (BE = Δ*E*_adduct_ – Δ*E*_acid-unit_ – Δ*E*_P-donor unit_), which helps in guessing the propensity of a given molecule to form a stabilizing adduct with phosphorene.

### Phosphorus–borane adducts

**Fig. 4:**
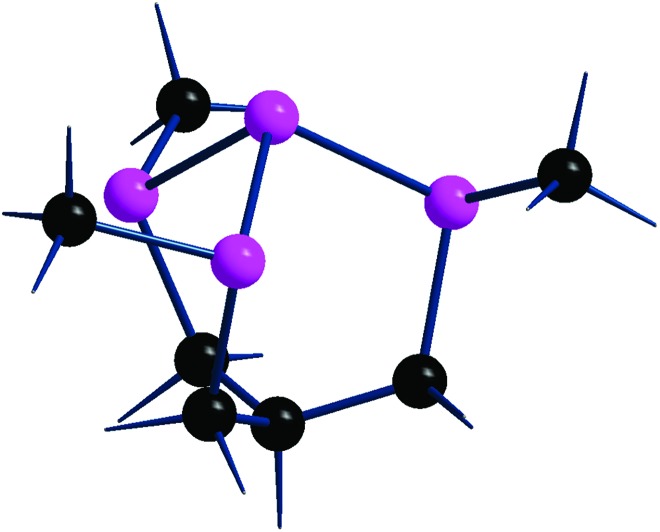
Structure of tris(di-*t*-butylphosphino)phosphane, P_3_P.^24^ The methyl groups of ^*t*^Bu substituents are omitted for clarity.

For comparative purposes, four different types of P_pyramidal_·BH_3_ adducts were modelled, the first two involving the molecular phosphines (CH_3_)_3_P and CH(CH_2_PCH_3_)_3_P. The latter with the acronym P_3_P,^24^ shown in Fig. 4, has a skeleton comparable with that of a local phosphorene subunit, and hence with four adjacent lone pairs. Although none of them directly points to another one, repulsion must be at work, perhaps even more effective than in the P_*n*_ species, where the interactions are spread throughout the entire 2D material.

The difference will be corroborated by a comparison of the P_3_P·BH_3_ adduct with the corresponding P_*n*_·BH_3_ ones, optimized for different borane coverages at a given side of the surface (see Table 1 and the associated discussion). Finally, a correlation is made with the white phosphorus adduct P_4_·BH_3_, where, at variance with P_3_P, there are three additional P–P bonds between the basal atoms of the molecule. In this case, the six edges of the P_4_ tetrahedron form a highly compact σ bonding system, to which also the four axial P lone pairs are contributing, given that the lowest lying P_4_ bonding combination of *a*_1_ symmetry largely consists of p_*z*_ in-pointing orbitals, and hence lies low in energy. As a consequence, the P_4_ basicity appears to be particularly low as previously pointed out by us.^12^

**Table 1:** Optimized P–B distances (Å), BE binding energies (eV) and band bap of various BH_3_ adducts of pyramidal P donors. In the case of phosphorene P_*n*_, different BH_3_ surface coverages have been considered

Model	P–B dist.	BE	Band gap
(CH_3_)_3_P·BH_3_	1.91	–1.61	
P_3_P·BH_3_	1.96	–1.15	
P_*n*_·0.031(BH_3_)	2.00	–0.60	2.32
P_*n*_·0.062(BH_3_)	2.00	–0.58	2.42
P_*n*_·0.125(BH_3_)	2.02	–0.51	2.61
P_*n*_·0.250(BH_3_)	1.98/2.11	–0.46	2.70
P_4_·BH_3_	2.08	–0.32	

In order to envisage some differences between the adducts, Table 1 reports optimized parameters such as the P–B distance, the BE binding energy and the band gap of the adduct. In particular, the (CH_3_)_3_P·BH_3_ one appears to be most stable in view of the shortest P–B distance of 1.91 Å (1.85 Å in some experimental structure)^25^ and the most negative Binding Energy (BE = –1.61 eV). P_3_P·BH_3_ is instead somewhat less stabilized, having a larger P–B distance of 1.96 Å and a less negative BE of –1.15 eV. The different basicity is not attributable to a lower energy of the pivotal P lone pair, given the similar destabilizing effects of either the C or P substituents. Rather, the reduced donor power toward the vacant B orbital is due to the minor contribution of the central P lone pair of P_3_P·(HOMO), which has instead about 20% larger mixing of the adjacent P lone pairs in spite of their somewhat unfavorable orientation.

Before addressing the behavior of phosphorene as a σ donor, it must be mentioned that the white phosphorus adduct P_4_·BH_3_ with P–B and BE values of 2.08 Å and –0.32 eV, respectively, represents the weakest adduct of the series. The reason for the very poor 2e^–^ donor power of P_4_ has already been outlined in our previous study of its demolition with I_2_, and indeed the highest barrier in the entire process leading to the final I_3_P product was encountered in the formation of the initial P_4_·I_2_ adduct.^12^ On the other hand, P_4_ is known to have residual donor power as suggested by some known η^1^ metal complexes, a first example being the trigonal bipyramidal species (NP_3_)Ni(η^1^-P_4_) species, reported long ago by our institute.^26^

**Fig. 5:**
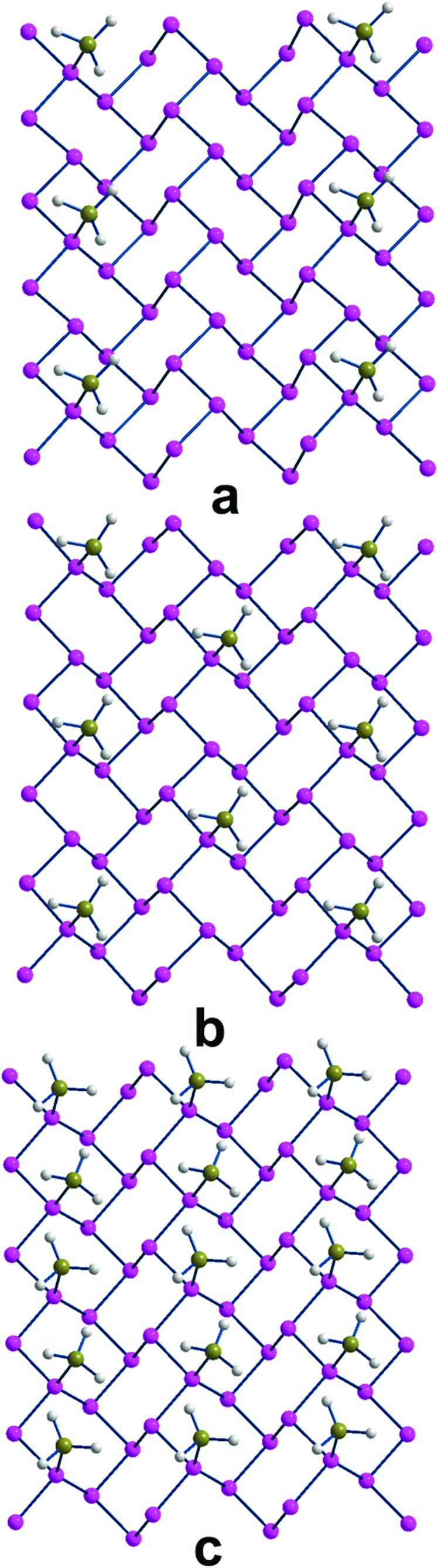
The three P_*n*_*x*(BH_3_) adducts with *x* = 0.062, 0.125 and 0.250 (a, b and c, respectively), corresponding to one BH_3_ molecule for every 16, 8 and 4 P atoms.

In the case of phosphorene itself, the P–B bonding strength determined for various P_*n*_·*x*BH_3_ adducts was systematically examined for different *x* values that each time doubled the density of borane on the surface. Fig. 5 shows a top view of the three progressively more covered species, which correspond to one BH_3_ molecule for every 16 and 8 and 4 P atoms of P_*n*_. Additionally, Table 1 reports the parameters also for the least dense adduct (ratio 1 : 32), where *x* corresponds to a coverage of about 3.125% *vs*. the other values of 6.25%, 12.50% and 25.0% in the series. Initially, the doubling of the borane coverage causes very small variations of the structural and energy parameters, because the added BH_3_ molecules do not significantly interfere with each other. Thus, the P–B distance of 2.0 Å remains unaffected and the BE is reduced by only 0.02 eV. In the subsequent passage from 16 to 8 P atoms for any BH_3_, the reduction of the P_*n*_ donor power is evident. In fact, the P–B distance elongates up to 2.02 Å and BE reduces to –0.51 eV.

Finally, in reaching the maximum BH_3_ density (25%), the system is evidently affected, also in view of the two short contacts between neighboring boranes. This determines also an asymmetry of the adjacent P–B distances (1.98 and 2.11 Å) while the difference in BE per BH_3_ unit becomes as small as –0.46 eV, confirming the increasing repulsion between the absorbates, no more tightly bound.

**Fig. 6:**
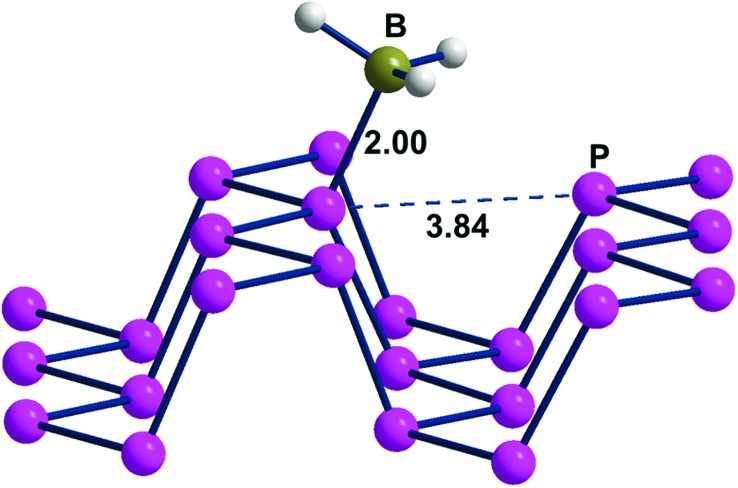
A portion of the P_*n*_·BH_3_ adduct, where 1 borane molecule is added to a supercell consisting of 16 P atoms.

Fig. 6, presenting a lateral view of the adduct with 6.2% BH_3_ coverage, highlights some P_*n*_ deformation. In particular, the channel on which the attached BH_3_ molecule projects is more broadened than the adjacent one by about 0.3 Å, thus allowing a larger cradle for the absorbed molecule(s). Recall that a similar broadening of the ditch was simulated for naked phosphorene by elongating the cell parameter *b*, as shown in Fig. 3. In that case, the gap between the frontier and conduction bands increased consistently with the results of Table 1 with a maximum 0.5 eV difference on increasing the BH_3_ density at the surface. From a qualitative electronic viewpoint, the larger separation of the facing lone pairs at the ditch determines decreased repulsions, the effect being further enhanced by the larger electron drifts into the added boranes. Finally, the narrowing of the lone pair band also implies a larger gap from the conduction band, which, being formed by the P–P σ* levels, is scarcely affected by these geometric perturbations.

### Phosphorus–diiodine adducts

The residual I_2_ acidity due to its low lying σ* level favors a collinear nucleophilic attack of an external base,^12^ for instance an axial lone pair of a P_pyramidal_ atom. At variance with the BH_3_ acceptor, two structural parameters may help in evaluating the donor–acceptor interactions with I_2_, namely a shrinking of the P–I linkage being formed and the corresponding I–I elongation due to the increased population of the I_2_ antibonding level. The mentioned geometric parameters together with the BE values are reported in Table 2 for the same series of P donors considered in Table 1. Starting with tri-alkyl phosphines, the nucleophilic attack on I_2_ is the most efficient, as proved by the experimental adduct ^i^Pr_3_P·I_2_ 23 and the optimized (CH_3_)_3_P·I_2_ one.^12^ In both cases, the P–I linkage is as short as ∼2.4 Å while the I–I distance is largely elongated (3.40 Å *vs.* the 2.73 Å value for the free diatomic). The persisting collinearity of the adducts recalls the classic Halogen Bonding (XB) picture with the expected asymmetric linkages.^27^ On the other hand, the attacked diatomic can be definitely split with the assistance of a second I_2_ molecule, which extracts the terminal iodide affording the ion pair [Pr_3_P_3_I]^+^ [I_3_]^–^. The corresponding distances in Table 2 are slightly less pronounced than those already determined by us through a different computational approach,^28^ while the present BE value of –0.98 eV is consistent with the previously calculated Δ*G* of –23.5 kcal mol^–1^ (–1.01 eV),^12^ which includes the penalizing entropy term. The latter should be almost constant for the whole series in Table 2. By switching to the phosphine adduct P_3_P·I_2_, the diatomic is somewhat less activated given the P–I and I–I distances of 2.86 and 3.03 Å, respectively. The point is also corroborated by the smaller BE value *vs*. (CH_3_)_3_P·I_2_ (–0.65 *vs*. –0.98 eV), implying a minor electron transfer from the P lone pair into I_2_ σ*. As stated above, the P_3_P weaker donor character is due to the about 20% mixing into the HOMO of the three lateral P lone pairs with the central one.

**Table 2:** Optimized P–I and I–I distances (Å) and the BE binding energies (eV) for the selected series of P_pyramidal_·I_2_ adducts analogous to those in Table 1. For simplicity, the uniquely examined P_*n*_ adduct contained one I_2_ molecule for any 16 P atoms at the surface

P_donor_	P_pyramidal_·I_2_		
	P–I	I–I	BE
(CH_3_)_3_P	2.78	3.07	–0.98
P_3_P	2.86	3.03	–0.65
P_*n*_	3.17	2.94	–0.18
P_4_	3.19	2.92	–0.13

The calculations confirm a very weak interaction in P_4_·I_2_, as suggested by the almost unperturbed geometries of the two molecular components and the very small –0.13 eV exothermicity.

In a previous study of the multi-step demolition of P_4_ with I_2_ to give I_3_P, the evolution from the first adduct P_4_·I_2_ was the most difficult one for the entire process in view of the high and unique barrier (Δ*G* = +14.6 kcal mol^–1^). In actuality, a second I_2_ molecule had to be involved as well as in all the subsequent steps.^12^ In this manner, two distinct P–I linkages could be formed in place of the pre-existing P–P bond, while a new I_2_ molecule was regenerated *in situ* being ready for subsequent reactivity. With this picture in mind, we explored whether also phosphorene could undergo any similar P–P cleavage under the action of two diatomics. Again we started with the XB type adduct P_*n*_·I_2_ where one diatomic interacts with a P_*n*_ cell of 16 P atoms with an activation that is only marginally larger than that in P_4_·I_2_ (differences in the P–I and I–I distances of about 0.2 Å and a slightly more exothermic energy balance of –0.18 *vs*. –0.13 eV). Upon the addition of a second I_2_ molecule, the optimized P_*n*_·2I_2_ model, reported in Fig. S1,† shows that the di-iodine activation only barely increases. In fact, the first added I_2_ molecule has P–I_1_ and I_1_–I_2_ variations no larger than 0.05 Å and the same result applies also to the second I_2_ one (see Table S1†). In this case, the overall BE value indicates an extra energy gain of only –0.12 eV, but more important, a further evolution of the system seems to be prevented by an unsuitable stereochemistry. While in the P_4_ case, it was evident that a remote I atom of the 2I_2_ grouping can potentially perform a nucleophilic attack into the P–P σ* level and induce its cleavage,^12^ the same does not apply to the 2D P_*n*_ species in view of the unsuitably oriented P–P bonds. Perhaps, the mechanism could be pursued for the high temperature transformation of the red phosphorus allotrope into the black one thanks to the involvement of I_2_ molecules generated by SnI_4_.^29^ In this case, the stereochemistry of the P–P linkages of red phosphorus is not as flat as in phosphorene, possibly favoring the action of I_2_ similarly to that proposed for P_4_. Obviously, the problem has to be tackled in some depth in order to corroborate such a conclusion.

### Transition metal fragments for covalent anchoring at phosphorene

Phosphorus based ligands are ubiquitous in organometallic chemistry and have potential relevance in catalytic processes. In principle, also one or more adjacent pyramidal P donors of phosphorene could coordinate an unsaturated metal center associated with a number of external coligands. This implies the covalent functionalization of the 2D material with metal centers, a subject scarcely tackled up to now at both the experimental and theoretical levels.^15^–^18^ The new P_*n*_–M bonding may, in principle, ensure full metal saturation, although a residual unsaturation may become relevant to support a catalytic behavior of the species anchored at the surface.

To choose the metal fragments which may be best suited for the phosphorene functionalization, it is first important to have a good understanding of the stereochemical and electronic properties of both the interacting phosphorene and transition metal fragment. In the choice of the latter, a leading concept is that of the *isolobal analogy*,^30^ which allows their tailoring to support single or multiple 2e^–^ interactions with neighbor P_*n*_ lone pairs. A general strategy is to start from a formally saturated metal complex, which, on losing one or more coligands, acquires a variable number of vacant σ hybrids acting as the acceptors of some phosphorene P lone pairs. The donor power of the latter has already been found to be particularly weak; hence it is important that the original metal complex carries some even weaker ligands to be eventually replaced. As another problem, the generated metal fragments should not create peculiar steric problems in the approach to the 2D material but also in their reorientation on the surface to maximize the σ overlap(s). Another important limitation in the choice of the metal fragment is its possible charge derived from the combination of different metals and coligands. In this case, the necessary electroneutrality of the solid state system imposes the presence of a counterion in close proximity to the charge metal fragment anchored at the surface. Clearly, for an optimal metal selection in the P functionalization, one should preferentially work with an uncharged metal fragment.

**Fig. 7:**
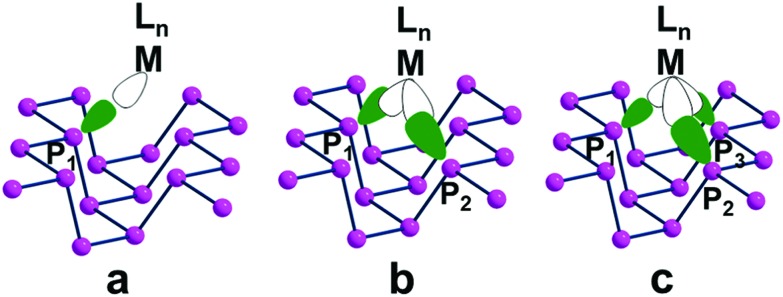
A phosphorene channel with differently anchored L_*n*_M fragments: (a) η^1^ coordination of P_*n*_ with a single σ acceptor metal; (b) η^2^ coordination at a doubly (*cis*) unsaturated metal fragment; (c) η^3^ coordination at a triply (*fac*) unsaturated metal fragment.

Fig. 7 shows top views of one phosphorene's face with differently hanging metal fragments. The latter differ in the number of σ hybrids stemming from the metal and interacting with one, two or three P_*n*_ lone pairs, as shown in Fig. 7a–c. Since any P lone pair forms an angle of about ∼30° with the P_*n*_ sheet, the unique P_1_–M linkage of the η^1^ coordination (**7a**) is expected to be bent on the surface, although in some cases some reorientation is observed (see below).

In the case of the η^2^ and η^3^ coordinations (**7b** and **7c**, respectively), not all the interactions imply an optimal orbital overlap. In the former case, for instance, the lone pairs at the P_1_ and P_2_ atoms, originally lying in parallel planes at the opposite sides of a ditch (see Fig. 1), need to interact simultaneously with a midway M atom. To maximize the overlap some local torsion should occur at the surface, which has however a very scarce degree of flexibility. A similar problem arises for the η^3^ coordination, which involves one atom at one bank of the ditch (P_1_), while the P_2_ and P_3_ ones are sequential at the opposite side. In this case, only the P_1_ lone pair can, in principle, attain an optimal overlap with one M σ vacant hybrid; conversely, those at the P_2_ and P_3_ ones should attempt a rather difficult reorientation. The problem will be later confirmed by the larger P_2_–M and P_3_–M bonding distances compared to the P_1_–M one.

### η^1^ coordination mode

**Scheme 1:**
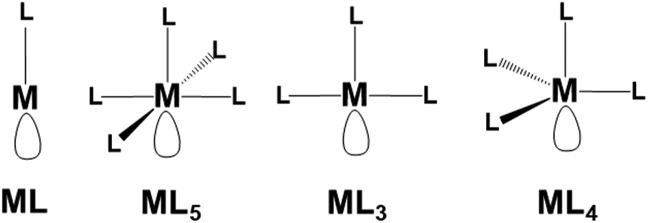
Generalized metal fragments in principle suited for the η^1^ coordination of phosphorene.

Possible examples of the general transition metal fragments featuring a singly vacant σ hybrid are shown in Scheme 1. Tentatively, we selected some general models to test in each case the possible P_*n*_ functionalization. Once again, we defined a cell of sixteen P atoms for each added metal fragment to test *in silico* the most relevant aspects of this chemistry.

#### d^10^-ML linear fragment

**Fig. 8:**
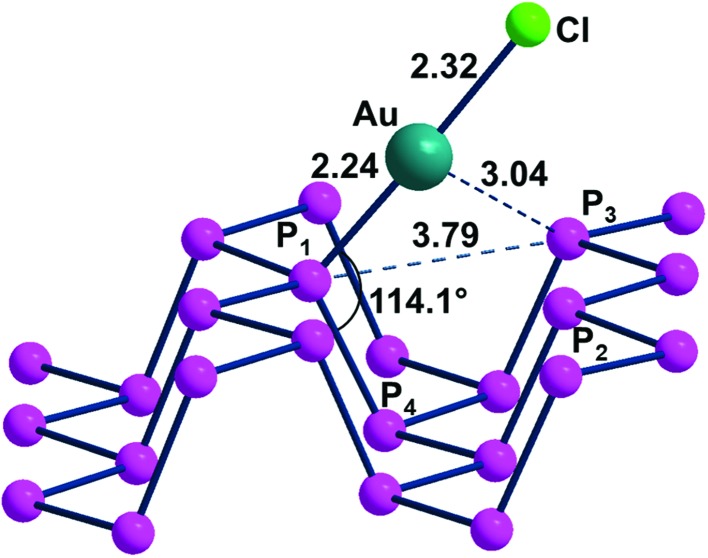
The optimized adduct ClAu(η^1^-P_*n*_).

The η^1^-P_*n*_ covalent functionalization was best verified using the d^10^-AuCl fragment. In this case, no major steric hindrance problem seems to be at work in the linear alignment of one P_*n*_ lone pair with the Au(i) vacant σ hybrid. The uncharged Au(i)Cl fragment can be, in principle, derived from a linear complex such as AuCl_2_^–^ (ref. 31) or ClAuL upon the extraction of a Cl^–^ anion or a L neutral ligand such as Me_2_S^32^ or R_3_P.^33^ Given the about 30° bias of any P lone pair on the average P_*n*_ plane, the P_4_–P_1_–Au angle of 114° of the optimized ClAu(η^1^-P_*n*_) adduct (Fig. 8) is close to the ideal 110° value, thus ensuring an almost optimal overlap between the metal and P σ orbitals. This point is corroborated by the 2.24 Å distance of the P_1_–Au bond, which is smaller than the sum of the covalent radii (2.43 Å)^34^ and close to the average single bond derived from Cambridge Database structures.^35^ Any major steric hindrance problem seems excluded by the large separation of about 3.85 Å between the terminal chloride ligand and the P_2_ and P_3_ atoms at the opposite bank of the channel. While the P_2_–Au and P_3_–Au distances are as large as 3.04 Å, it cannot be excluded that a residual attraction is at work.^36^ An indication may be the approximately 2° deviation from linearity of the P_1_–Au–Cl angle and the consequent biasing of the metal toward the P atoms at the other side of the ditch. The latter is about 0.2 Å widened with respect to free phosphorene, suggesting that the channel adapts for better accommodation of the projecting fragment, with an effect on the phosphorene band structure. The specificity of the latter in this case will be discussed below.

The stabilization of the ClAu(η^1^-P_*n*_) adduct is proved by the large BE value of –2.4 eV, which is about four times larger than the –0.58 eV value obtained for the non-metal species P_*n*_ BH_3_ in Table 1. In view of these results, it emerges that an opportune transition metal fragment can be one of the best candidates for the covalent functionalization of phosphorene. For an even more realistic energetic picture, the ClAu(η^1^-P_*n*_) adduct has been obtained through two alternative paths. Eqn (1) involves the precursor ClAu(Me_2_S), which first loses Me_2_S with a cost of +2.2 eV; hence the final energy balance is exothermic by –0.2 eV. Remarkably, the adduct formation from the other mentioned complex ClAu(Me_3_P) is more difficult, since eqn (2) is endothermic by +0.44 eV. Thus, phosphorene functionalization can be generalized to proceed from reactants carrying a good leaving group.

1 ClAu(Me_2_S) + P_*n*_ → ClAu(η^1^-P_*n*_) + Me_2_S

2 ClAu(Me_3_P) + P_*n*_ → ClAu(η^1^-P_*n*_) + Me_3_P

It is worth mentioning that previous computational studies on gold-phosphorene were reported for a system with single Au(0) atoms dispersed on the surface and η^3^ locally bound to the cavity formed by the P_1_, P_2_, P_3_ atoms.^16^ The average optimized Au–P distance of about 2.37 Å and the –1.61 eV stabilization energy seemed to confirm the propensity of gold toward P_*n*_ functionalization but the peculiar electronic nature of the Au(0) carrying an unpaired spin each was not specifically illustrated.

At this point, it is worth mentioning that, based on the *in silico* predictions about the ClAu(η^1^-P_*n*_) adduct, some experimental attempts were made by us to obtain a functionalized phosphorene surface, starting from the ClAu(Me_2_S) reactant. Unfortunately, we are still unable to present here any unquestionable result, because ClAu(Me_2_S) easily promotes the formation of gold nanoparticles.^37^ During the reaction, we indeed observed by-products, some of which have been spectroscopically characterized. The work, which is still in progress, will hopefully provide some reasonable explanation of the Au(i) → Au(0) reduction. To avoid the latter, we are trying to use some Au(i) linear complex other than ClAu(Me_2_S) for the P_*n*_ functionalization.

**Fig. 9:**
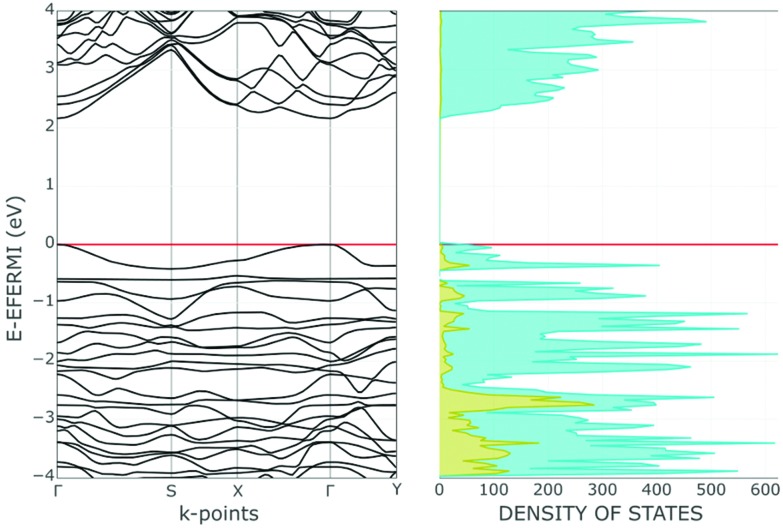
Band and DOS structures of the adduct of ClAu fragments to phosphorene (1 : 16 ratio). Near the Fermi level (red line), a significant contribution from gold d orbitals in yellow falls in between the valence and conducting bands with a consequent narrowing of band gaps.

Concerning the anticipated effect on the P_*n*_ band structure induced by the functionalization with ClAu, we noticed a decrease of the band gap, rather than the increase expected for the widening of some ditch. Indeed, the 2.26 eV band gap of free phosphorene becomes 2.17 eV in ClAu(η^1^-P_*n*_). A relatively easy explanation of the result emerges from the plots of the whole band and the corresponding Density of States (DOS) in Fig. 9.

Evidently, the highest portion of occupied bands (Fermi region) largely consists of a set of Au d orbitals (yellow projection), which are implicitly repulsive with P lone pairs. Importantly, the spread of the basic band structure determines a consequent reduction of the gap with respect to the conduction band. This point is further corroborated by a plot of the Crystal Overlap Orbital Population (COOP in Fig. S2†), which properly highlights the triggered d/P lone pair repulsions.

#### d^6^-ML_5_ square pyramidal (SP), d^8^-ML_3_ T-shaped and d^8^-ML_4_ trigonal pyramidal (TP) metal fragments

**Fig. 10:**
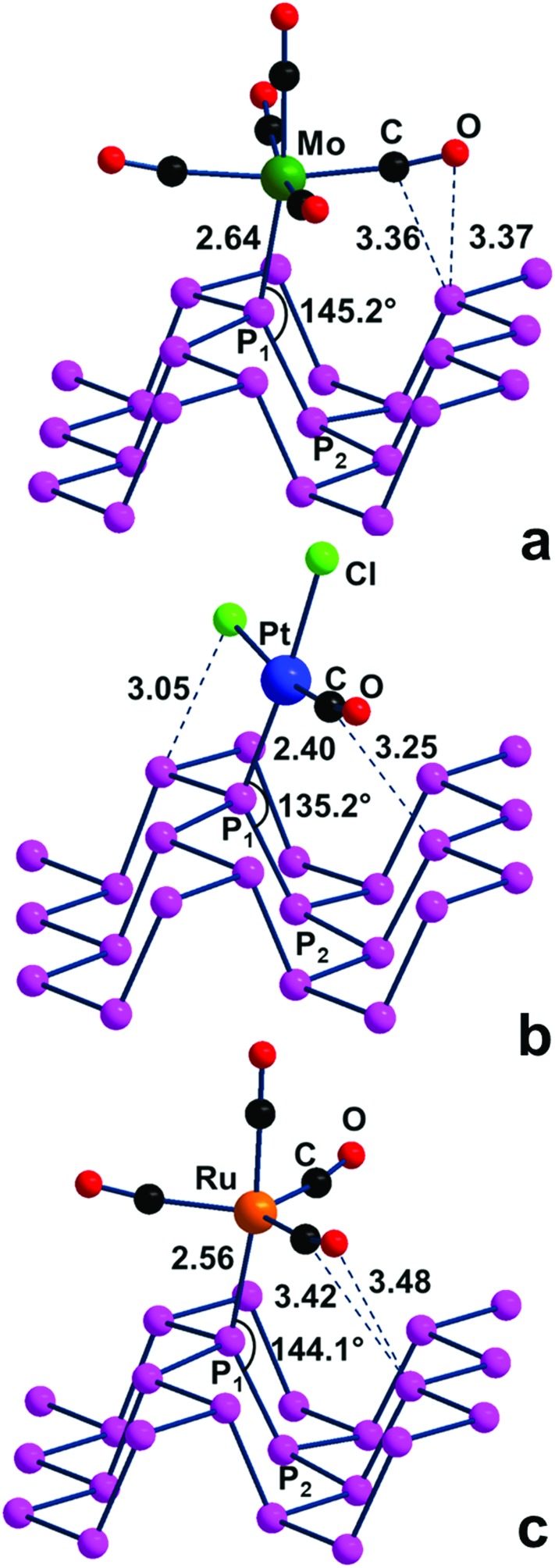
Optimized adducts between P_*n*_ and different metal fragments with a single σ acceptor function: (a) d^6^-(CO)_5_Mo Square Pyramid, SP; (b) d^8^-Cl_2_(CO)Pt T-shaped fragment; (c) d^8^-(CO)_4_Ru trigonal pyramid (TP).

The behavior of the additional metal fragments in Scheme 1, all carrying a uniquely vacant σ hybrid to be potentially exploited in the η^1^-P_*n*_ coordination, is briefly addressed here. For instance, a d^6^-ML_5_ SP fragment derived from a classical octahedral complex of group VI (M(CO)_6_ with M = Cr, Mo, W)^38^ may potentially interact with a P lone pair of the P_*n*_ surface upon some bending on the surface, which maximizes the σ overlap. In this case, however, close contacts are formed between P atoms and at least two basal CO ligands of the metal fragment with consequent steric effects. Something analogous occurs for a T-shaped fragment such as PtCl_2_(CO), which formally descends from the square planar d^8^ complex PtCl_2_(CO)_2_ 39 or a Trigonal Pyramidal (TP) one (*e.g.*, d^8^-(CO)_4_Ru) derived from a TBP precursor such as (CO)_5_Ru^40^. In actuality, all the mentioned P_*n*_ adducts were optimized, but in their structures, shown in Fig. 10a–c, the metal fragment is not bent but almost upright on the surface. This is confirmed by the P_2_–P_1_–M angles, which are about 25–35° more open than the ideal 110° value, hence far from allowing maximum σ overlap. Consequently, the P_1_–M bonds are weaker than expected, as confirmed by the optimized (M = Mo, Pt, Ru) distances, which are in general about 0.1 Å larger than single bonds.^35^ Most likely, the adopted geometry helps prevent short contacts between some coligand and P atoms on the surface. On the other hand, all of the structures of Fig. 10 are actual minima, which imply a certain degree of stability in spite of their scarce P–M direct bonding. A possible explanation may be based on the pointed out short vector(s) between some surface P atoms and the upper CO ligand. Remarkably, the latter is not exactly collinear with the bound metal atom but features an M–C–O angle bent up to about 15°. This can be suggestive of an incipient non-covalent P···C(O) interaction, occurring between a P lone pair and a vacant CO π* level mainly at the side of the C atom. Even weaker interactions of this type are likely at work in the adsorption of gaseous CO on phosphorene, as suggested by other authors.^14^ Since points like this are not in line with the presently studied covalent functionalization of phosphorene, the electronic features of the intriguing compounds of Fig. 10 have been no further explored.

### Poly-hapto coordination of a metal by phosphorene

The phosphorene's sequence of interlinked P atoms, each carrying a single σ lone pair, suggests the possibility of bis-chelate or tris-chelate behaviour of the 2D material. The functionality of phosphorene as a multiple donor must be related to the disposition of the selected close lone pairs at the P_1_, P_2_ and P_3_ atoms, as outlined in Fig. 7. Again, the identification of suitable metal fragments able to accept simultaneously two or three 2e^–^ donations can be gained from the application of the *isolobal analogy* concept.^30^

### η^2^ coordination mode

**Fig. 11:**
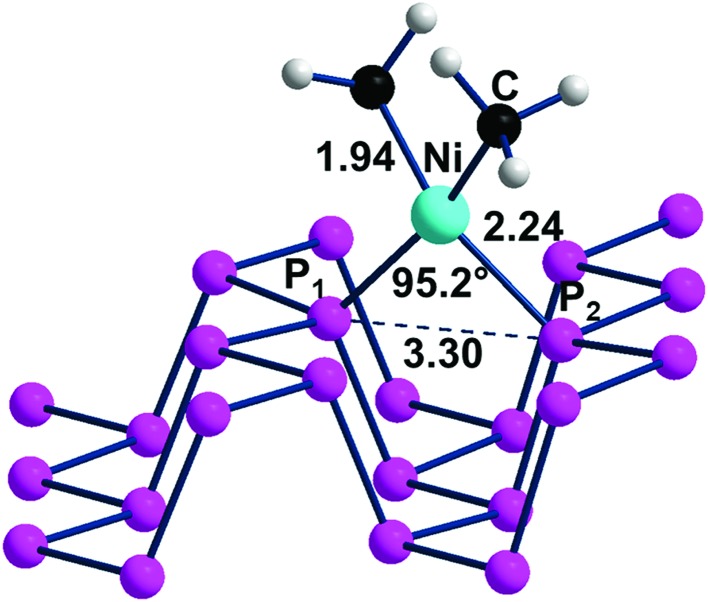
Optimized structure of the adduct (CH_3_)_2_Ni(η^2^-P_*n*_) with a Ni : P ratio of 1 : 16.

L_2_M fragments, derived from square planar d^8^ complexes upon removal of two uncharged *cis* ligands, carry two vacant σ hybrids suitable for the η^2^ coordination of phosphorene across one of the channels. For instance, a suitable precursor can be a Ni(ii) complex with two methyl ligands and two uncharged 2e^–^ donors (*e.g.*, H_2_O), which, on losing the water molecules, allows the orbital interactions of Fig. 7b in the product (CH_3_)_2_Ni(η^2^-P_*n*_). An optimization of the latter species, using again the 1 : 16 Ni : P ratio, confirmed that the P_1_ and P_2_ atoms across a P_*n*_ channel can also complete the square planar coordination of the metal as in the structure of Fig. 11. Here, the optimized Ni–P distances of 2.24 Å are about 0.1 Å longer than those in the comparable X-ray structures formed by a diphosphine chelate,^41^ while the P_1_–Ni–P_2_ angle of 95.2° is about 8° more open. These aspects are likely the consequence of the already known weaker donor power of the phosphorene P atoms and their strained geometry.

**Scheme 2:**
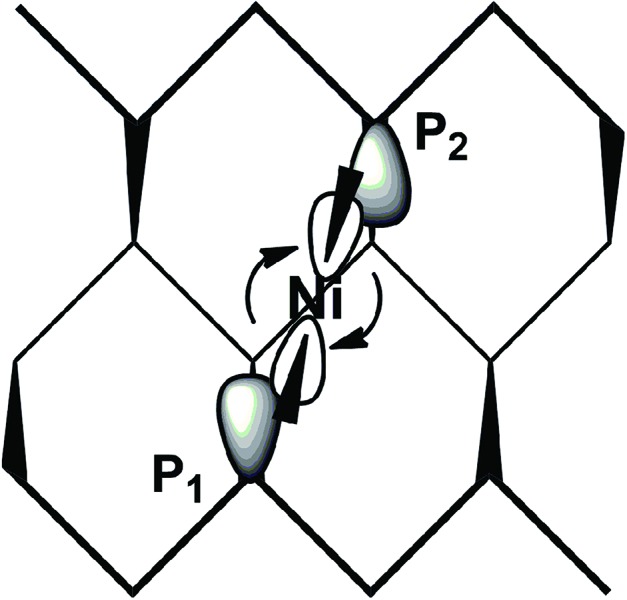
The suggested rearrangement of a L_2_Ni(ii) fragment across one P_*n*_ channel to maximize σ overlap.

The simultaneous coordination of the two P atoms at the opposite sides of a channel imposes a shrinkage of the latter, which is indicated by the 0.2 Å shorter P_1_···P_2_ separation compared to naked phosphorene. Correspondingly, the adjacent channel is somewhat widened. The P_1_ and P_2_ lone pairs involved in the η^2^-P_*n*_ coordination lie, as known, in parallel planes, and hence do not naturally converge into the unique metal position with nonoptimal overlap with its vacant σ hybrids. For this, the metal fragment is rotated by about 25° with respect to the perfect square planar geometry. Scheme 2 helps in understanding the orbital underpinning of the distortion, needed to improve the σ overlaps.

Through some minor deformation of the phosphorene surface and the indicated metal rearrangement, the obtainment of the final P_*n*_ adduct is indeed exothermic by –0.32 eV, as estimated from the reaction in eqn (3).

3 (CH_3_)_2_Ni(H_2_O)_2_ + P_*n*_ → (CH_3_)_2_Ni(η^2^-P_*n*_) + 2H_2_O

The quasi square planar coordination of d^8^-Ni(ii) on P_*n*_ determines an increase of the band gap with respect to that of naked phosphorene (from 2.26 to 2.31 eV, as shown in Fig. S3†). The trend is the opposite compared to that of the ClAu derivative. There must be a compromise between the reduced repulsion between the P_1_ and P_2_ lone pairs participating in metal binding and the larger repulsion between the non-involved ones due to the shrinking of the channel.

**Fig. 12:**
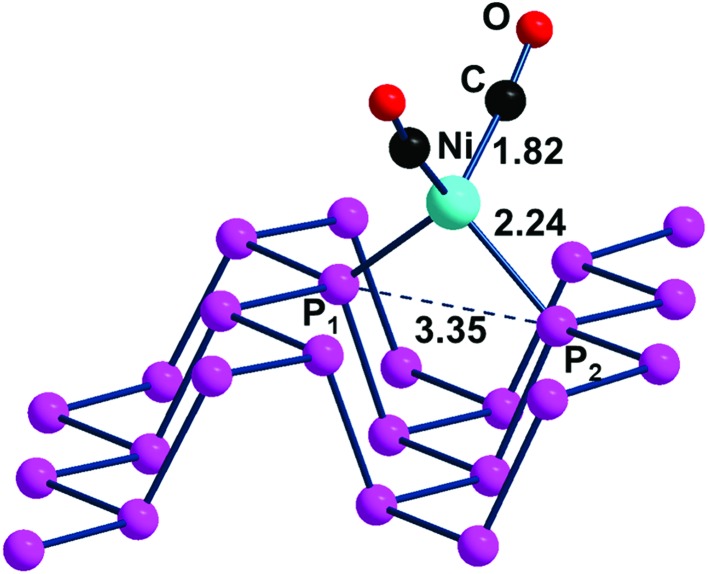
Optimized structure of the (CO)_2_Ni(η^2^-P_*n*_) adduct.

The η^2^ coordination of phosphorene may be potentially attained also by using alternative fragments. One of them can still be of the L_2_M type with a d^10^ rather than a d^8^ metal atom, which has as a precursor an 18e^–^ tetrahedral complex (T_d_). In this case, the two vacant σ hybrids of the L_2_M fragment lie high in energy for having exclusive s/p character without any d contribution. In general, the known Ni, Pd, Pt tetrahedra are stabilized by strong σ donors such as four COs^42^ or 2COs + 2R_3_P ligands.^43^,^44^ Since some complex is known with a dinitrogen chelate,^45^ we used the species (CO)_2_Ni(NH_3_)_2_ as a model reactant for the reaction with P_*n*_, reported in eqn (4). From the optimized adduct (CO)_2_Ni(η^2^-P_*n*_), having a local tetrahedral geometry as shown in Fig. 12, the process is estimated to be exothermic by –0.17 eV.

4 (CO)_2_Ni(NH_3_)_2_ + P_*n*_ → (CO)_2_Ni(η^2^-P_*n*_) + 2NH_3_

In the tetrahedral geometry of (CO)_2_Ni(η^2^-P_*n*_), the Ni(0)–P distances of 2.24 Å are close to those of the Ni(ii) square planar species of Fig. 11. Therefore, the channel width is again shortened by about 0.2 Å but the 2.22 eV band gap of the d^10^ adduct is now only slightly smaller than the 2.26 eV value of free phosphorene *vs*. the 2.31 eV value of the Ni(ii) case. Fig. S4† shows trends similar to those of the ClAu adduct in view of the position of the metal d orbitals in the highest portion of the valence band. A final piece of information concerns the approximately 70 cm^–1^ blue shift of the CO ligands found in the computed IR spectrum of (CO)_2_Ni(η^2^-P_*n*_) compared to that of the molecular model (CO)_2_Ni(PMe_3_)_2_. This feature is not surprising given the known weak donor power of the 2D material, with a consequently reduced back donation from the metal into CO ligands.

Another metal fragment for potential η^2^-P_*n*_ coordination is a d^6^-L_4_M butterfly, obtained upon removal of two *cis* equatorial ligands from a d^6^-L_6_M octahedron. A possible example is (CO)_4_M (M = Cr, Mo and W), obtainable for instance from an octahedron with two additional dialkyl sulphide ligands.^46^,^47^ Optimization of the (CO)_4_Mo(η^2^-P_*n*_) adduct (see Fig. S5†) shows that the Mo–P interactions are relatively weak in view of the 2.56 Å distances and other evident distortions of steric origin. The scarce propensity of the butterfly (CO)_4_Mo fragment to support P_*n*_ di-hapto coordination is corroborated by the large +1.02 eV endothermicity of (CO)_4_Mo(η^2^-P_*n*_), as estimated from eqn (5). Perhaps in general, phosphorene functionalization is unlikely with a butterfly fragment.

5 (CO)_4_Mo((CH_3_)_2_S)_2_ + P_*n*_ → (CO_4_)Mo(η^2^-P_*n*_) + 2(CH_3_)_2_S

### η^3^ coordination mode

The contiguous P_3_ triangles at the P_*n*_ surfaces (Fig. 1) are attained by involving a single and a pair of P atoms belonging to different zigzag chains. The triangles are isosceles but not dramatically far from being equilateral (3.62 and 3.35 Å for the two across-channel sides and the in-chain one). In principle, the three atoms in question represent an ensemble of *fac* ligands at a d^6^-L_6_M octahedron, whose other three vertexes can be those of a L_3_M fragment, such as the (CO)_3_Mo one of the complex (CO)_3_Mo(η^6^-*p*-xylene),^48^ whose poly-hapto aryl ring can be easily removed.

**Scheme 3:**
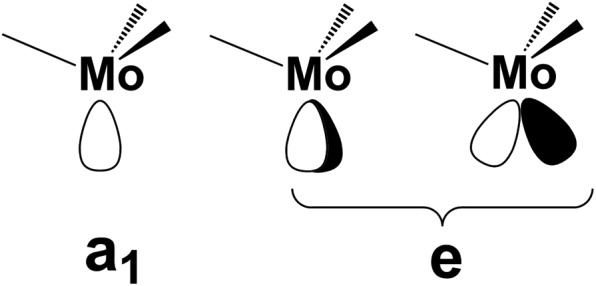
Vacant FMOs of the uncharged (CO)_3_Mo fragment with their corresponding symmetries.

From the orbital interaction viewpoint, the unsaturated trigonal pyramidal (TP) (CO)_3_Mo fragment has the three vacant and delocalized MOs of a_1_ + e (degenerate) symmetries, shown in Scheme 3. These can match with phosphorene's lone pair combinations to allow η^3^-P_*n*_ coordination.

**Fig. 13:**
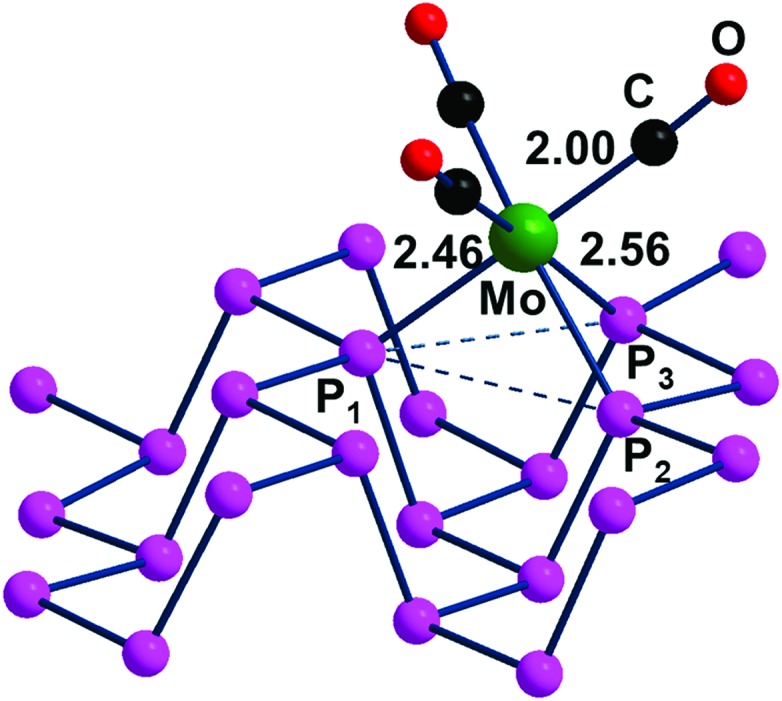
Optimized structure of the adduct (CO)_3_Mo(η^3^-P_*n*_).

However, the e symmetry matchings are not fully equivalent due to the natural isosceles shape of the P_3_ triangle on the surface. In fact, the optimized (CO)_3_Mo(η^3^-P_*n*_) adduct of Fig. 13 has a P_1_–Mo bond of 2.46 Å, which is about 0.1 Å shorter than the P_2_–Mo and P_3_–Mo ones of 2.56 Å.

**Scheme 4:**
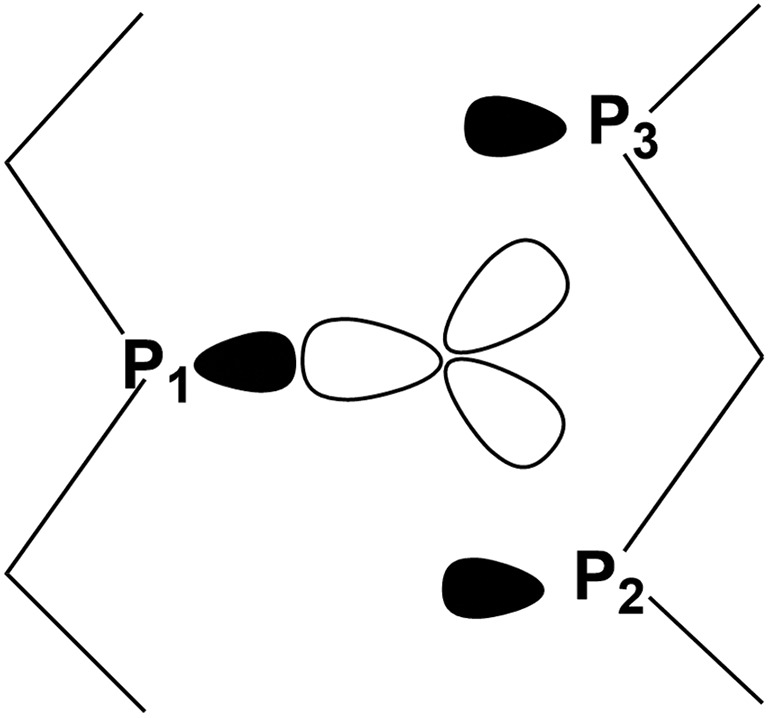
Disposition of the vacant metal σ hybrids and the P_1_, P_2_ and P_3_ lone pairs in a potential tri-hapto coordination.

As indicated by Scheme 4, the P_1_ lone pair is properly oriented for optimal MO interactions with both a_1_ and the e orbitals of Scheme 3. Conversely, the P_2_ and P_3_ lone pairs are not equally well oriented and can hardly rearrange due to the P_*n*_ skeleton rigidity. Eventually, however, the divergences in bonding are not so dramatic; hence an essential tri-hapto coordination of phosphorene may be assumed.

From the energy viewpoint, the η^3^-P_*n*_ formation is exothermic by about –0.33 eV, as estimated by the reaction in eqn (6), which proceeds from the precursor (CO)_3_Mo(η^3^-*p*-xylene).

6 (CO)_3_Mo(η^3^-*p*-xylene) + P_*n*_ → (CO)_3_Mo(η^3^-P_*n*_) + *p*-xylene

Incidentally, the scarce deformation of the coordinated P_*n*_*vs*. the free 2D material has a cost of +0.28 eV, as it results from a single point calculation of the final adduct upon removal of the (CO)_3_Mo fragments. Evidently, the formed P–Mo bonds almost double the energy lost on the P_*n*_ deformation. In addition, the discrete complex (CO)_3_Mo(PMe_3_)_3_ was optimized to compare its computed IR spectrum with that of (CO)_3_Mo(η^3^-P_*n*_). For consistency, the vibrational frequencies of all species were calculated by using the same CRYSTAL software package.^19^ Once again, the donor power of phosphorene is found to be scarce as indicated by the 60 cm^–1^ blue shift of the CO stretching in the adduct *vs*. those of the complex (CO)_3_Mo(PMe_3_)_3_. Consider that in the latter the carbo-substituted phosphines have high donor power, so that the larger electron density at the metal allows larger back-donation with different IR responses for CO stretching. As another point, adducts of the type L_3_Mo(η^3^-P_*n*_) with three single bulky substituted phosphines^49^ or a common tripodal chelate such as CH_3_C(CH_2_)_3_PPh_2_ 50 are unlikely because of the steric hindrance problems arising when the substituents point down on the P_*n*_ surface.

Finally, as already done for the P_*n*_ η^1^ coordination with the ClAu fragment, we examined how the electronic structure and the determining band gap may be influenced by the (CO)_3_Mo functionalization. The corresponding gap of 2.34 eV for (CO)_3_Mo(η^3^-P_*n*_) is larger than that in free phosphorene and the discussed ClAu(η^1^-P_*n*_) adduct (2.26 and 2.17 eV, respectively). At variance with the latter case, the P lone pairs, which participate in the P–Mo bonding, lose their reciprocal repulsive character, with a lowering of the valence band spread, and a consequent increase of the band gap. On the other hand, the right side of Fig. S6† shows that in both the valence and conduction bands, the π interactions between the t_2g_ metal d orbitals and CO π* levels have a significant contribution. In particular, the stabilized Mo d orbitals represent the highest limit of the valence band, while the opposite occurs for ClAu(η^1^-P_*n*_), where the repulsions between the low lying and filled Au d orbitals and the P lone pairs fix the band gap.

## Conclusions

This article has addressed potential cases of phosphorene's covalent functionalization, stemming from the compactness of the P lone pairs at the surface. Their behavior as single or combined 2e^–^ donors has been underlined concerning the interactions with acidic main group units or unsaturated transition metal fragments. Interesting electronic aspects have emerged from the solid state calculations compared with related molecular models. All the interactions are of the acid–base Lewis type, even though the P lone pairs of the P_*n*_ surface feature limited donor power. A possible reason for this is that the lone pairs participating in the valence bands are not separated but somewhat mixed into each other throughout the surface. Accordingly, such a delocalization does not allow maximized σ overlap with the added acidic groups. Note that in this case the invoked delocalization is very different from the π one in planar graphene, with determining π bonding to be excluded for phosphorene. These points have clearly emerged for instance from the adducts with the acidic BH_3_ molecule and, in particular, those having variable coverage of the surface. Di-iodine has also been tested to check whether it can induce P–P cleavage at phosphorene, as we recently found for white phosphorus (P_4_).^12^ This hypothesis had to be excluded, because I_2_ (or two combined molecules of it) in no case has the possibility of attacking from outside any P–P σ* level with consequent P–P cleavage.

Next, we switched to various transition metal fragments featuring a variable number of vacant metal lobes, based on the *isolobal analogy* concept.^30^ Accordingly, we examined the possibility that some given metal acceptor interacts with one or more neighbor P_*n*_ lone pairs and affords its covalent functionalization. M–P_*n*_ derivatives of various hapticities were optimized, providing useful information on stereochemistry and energy. Evident steric hindrance problems emerge even when a metal fragment is electronically suited but has an unsuitable disposition of the coligands, in particular if bulky. Other derived properties of the adducts have been examined such as the vibrational spectra of the CO coligands, when present. Another tackled point concerns the band gap of the functionalized adduct *vs*. that of naked phosphorene as a consequence of the variable repulsion between P lone pairs. A first example of perturbation is given by the stacking of the P_*n*_ sheets to reform black phosphorus. Only ten P_*n*_ sheets are sufficient to reproduce the much smaller band gap of the 3D material, because the bilateral repulsions between the sheets quickly spread the frontier band and reduce its separation from the conduction band. On the other hand, the functionalization of a single P_*n*_ face has contrasting and discussed effects such as the widening of the ditches on associating acidic groups, which in turn reduces the repulsion between lone pairs. In the case of metal coordination, its d orbital set may fall at the top of the bands formed by P lone pairs with consequent modification of the ultimate band gap. Qualitative explanations have been proposed also for more complex cases.

While our analysis has indicated various possibilities of covalent functionalization of phosphorene with acidic groups, the corresponding experimental evidence remains scarce. Certainly, one reason is the difficult isolation and manipulation of the 2D material, given its known sensitivity to external agents such as oxygen or humidity. A preliminary elimination of the latter is fundamental to perform new useful chemistry. In any case, we trust that our models may be later exploited by some brave experimentalist to construct viable species and open new application gates in the chemistry of the important 2D material.

## Computational details

The optimized geometries and energetics of the naked and functionalized phosphorene surface have been established at the B3LYP-DFT^21^ level of theory by using the CRYSTAL17 software package.^19^ A selected supercell of 16 phosphorus atoms has been usually adopted for performing the optimization of both the atomic positions and lattice parameters. The TZVP basis set^51^ has been used for all the atomic species. It is only for the elements Ru,^52^ Mo,^53^ for Au^54^ and I^55^ that different *pseudo-potentials* have been employed. In the metal adducts with CO ligands, the vibrational frequencies have been carried out to compare the donor power of the P_*n*_ surface with those of other P donors. Band and Crystal Overlap Orbital Populations (COOP) analyses have been carried out with the available routines of the CRYSTAL17 software package.^19^ A list of all the optimized structural and energy parameters is available in the ESI.†

## Conflicts of interest

There are no conflicts to declare.

## Supplementary Material

Supplementary information
