# Supplementary material for: Modelling strategies for the covalent functionalization of 2D phosphorene
Source: Dalton Trans. 2018 Nov 12;47(48):17243–56. doi: 10.1039/c8dt03628d (PMC6301276; doi:10.1039/c8dt03628d)
Supplement: Supplementary file 1 [file DT-047-C8DT03628D-s001.pdf]

Electronic Supplementary Material (ESI) for Dalton Transactions.
This journal is © The Royal Society of Chemistry 2018

Modelling Strategies for the Covalent Functionalization of 2D Phosphorene

Andrea Ienco,* Gabriele Manca,* Maurizio Peruzzini and Carlo Mealli

Istituto di Chimica dei Composti Organometallici – Consiglio Nazionale delle Ricerche (CNR-ICCOM), Via Madonna del Piano 10, 50019, Sesto Fiorentino (FI), Italy.

Supporting Information

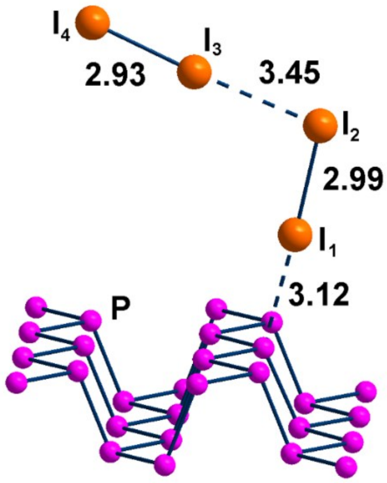

Figure S1. Optimized structure of $P_n \cdot 2I_2$ adduct.

Table S1. Geometric and energetic values of the optimized $P_{pyram} \cdot 2I_2$ species. The BE values are relative to formation of the adducts between P_{pyram} and two I_2 molecules. The $[P_{pyram}I]^+ [I_3]^-$ ion pair formation appears plausible only for the stronger phosphine donors.

	$P_{pyram} \cdot 2I_2$ adduct				
	P-I_1 (Å)	I_1-I_2 (Å)	$I_2 \cdots I_3$ (Å)	I_3-I_4 (Å)	BE (eV)
$(CH_3)_3P \cdot 2I_2$	2.59	3.22	3.30	3.01	-1.52
$P_3P \cdot 2I_2$	2.68	3.13	3.34	2.98	-1.20
$P_n \cdot 2I_2$	3.12	2.99	3.45	2.93	-0.32
$P_4 \cdot 2I_2$	3.19	2.95	3.50	2.92	-0.28

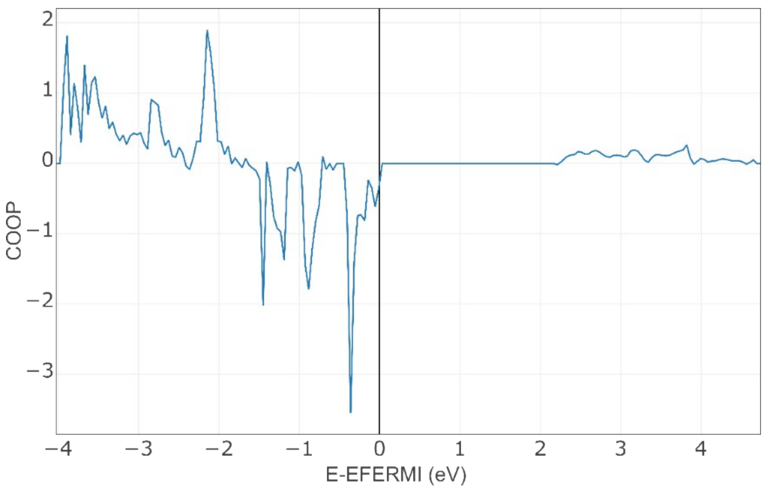

Figure S2. Crystal Overlap Orbital Population (COOP) between Au P_3 atoms in ClAu (η^1-P_n) adduct.

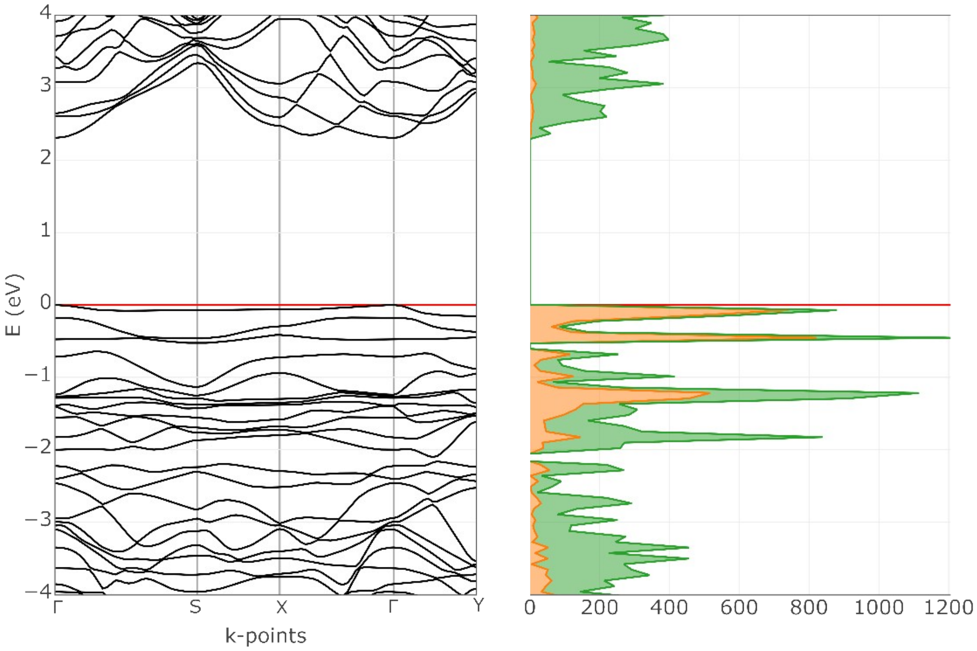

Figure S3. Band and DOS structure for the adduct of phosphorene with $(CH_3)_2Ni$ fragments. The contribution from the metal fragment is highlighted in orange.

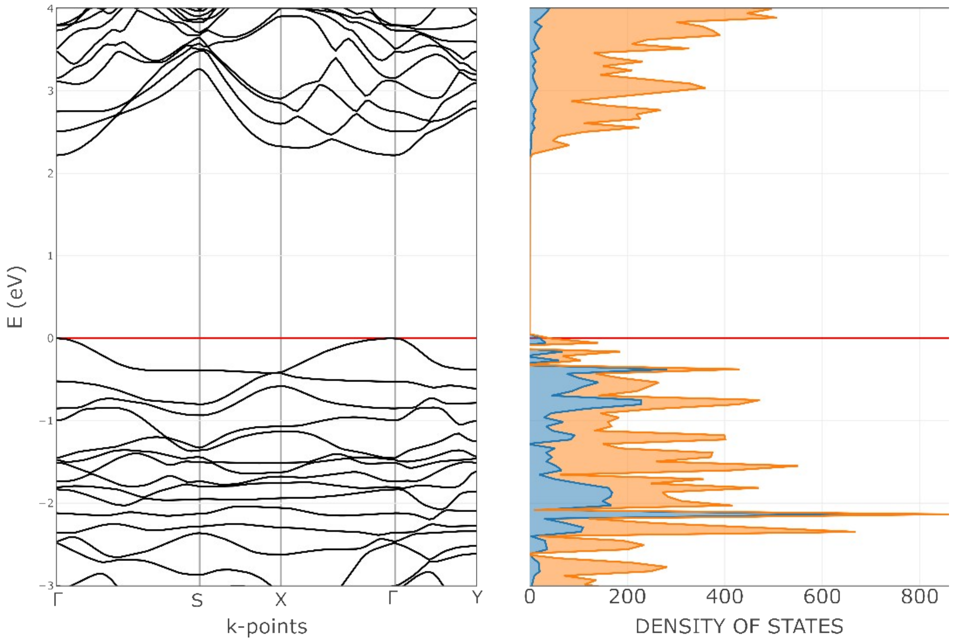

Figure S4. Band and DOS structure for the adduct of phosphorene with $(CO)_2Ni$ fragments. The contribution from the metal fragment is highlighted in blue.

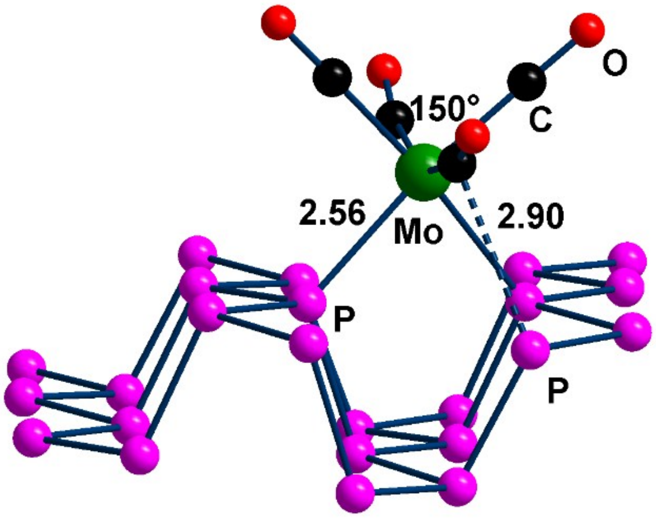

Figure S5. Optimized structure of the $(CO)_4Mo(\eta^2\text{-}P_n)$ adduct.

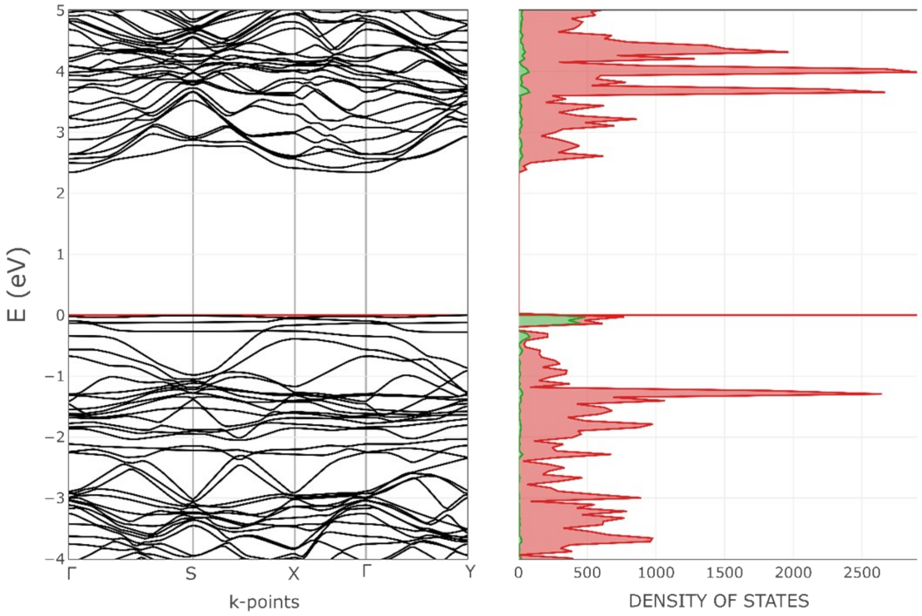

Figure S6. Band and DOS structure for the adduct of phosphorene with $(CO)_3Mo$ fragments. The contribution from the metal fragment is highlighted in green.

Geometric and Energy parameters of optimized Structures

$(CH_3)_3P$

Sy	X	Y	Z
P	0.007911	-0.498916	1.867526
C	1.666397	-1.337860	1.926431
H	2.285333	-1.120384	1.054730
H	2.209058	-1.025361	2.818121
H	1.534263	-2.417442	1.991692
C	-0.507292	-1.010913	0.155717
H	-1.414184	-0.480559	-0.133247
H	0.258530	-0.815393	-0.596330
H	-0.738188	-2.075677	0.140331
C	0.578668	1.229232	1.485923
H	1.072559	1.658054	2.357366
H	1.270548	1.273397	0.643543
H	-0.278904	1.861621	1.258096

Energy: -460.9874835821 Hartrees

P_3P

Sy	X	Y	Z
P	-0.575122	1.881450	-0.983271
P	0.000947	0.000112	0.098024
C	-0.602601	1.348448	-2.794600
H	-1.649337	1.373768	-3.101565
H	-0.110304	2.150178	-3.348604
C	0.000165	-0.000374	-3.202170
H	0.000129	-0.000589	-4.299772
H	0.745993	2.424824	-0.965624
P	-1.342450	-1.438412	-0.982688
C	-0.866576	-1.196624	-2.794044
H	-0.365287	-2.116006	-3.100552
H	-1.807052	-1.171321	-3.348014
C	1.469707	-0.152343	-2.794399
P	1.917752	-0.445010	-0.983577
H	1.721977	-1.859891	-0.966714
H	2.014123	0.742856	-3.099474
H	1.918905	-0.978330	-3.349472
H	-2.470969	-0.562935	-0.965184

Energy: -1523.603867883 Hartrees

P_4

Sy	X	Y	Z
P	0.000001	-0.000000	-1.354881
P	0.638698	1.106254	0.451623
P	0.638699	-1.106254	0.451623
P	-1.277388	0.000000	0.451624

Energy: -1365.273533907 Hartrees

P_n

a	b	c	alpha	beta	gamma
6.6993	9.4259	1.0000	90.0000	90.0000	90.0005

Sy	X	Y	Z
P	0.208734	0.054717	1.291381
P	-0.041272	0.212801	1.290651
P	-0.041226	0.304562	-0.828676
P	0.208748	0.462693	-0.829171
P	-0.291262	0.054721	1.291174
P	0.458741	0.212803	1.290938
P	0.458714	0.304565	-0.828434
P	-0.291255	0.462630	-0.829452
P	0.208737	-0.445534	1.290205
P	-0.041278	-0.287460	1.292008
P	-0.041268	-0.195405	-0.826308
P	0.208747	-0.037338	-0.826986
P	-0.291250	-0.445575	1.289761
P	0.458769	-0.287470	1.292235
P	0.458739	-0.195422	-0.826045
P	-0.291273	-0.037329	-0.827043

Energy : -5461.168208963 Hartrees

BH_3

Sy	X	Y	Z
B	0.567328	0.202859	3.281106
H	1.705750	0.271616	2.976713
H	0.079082	-0.847936	3.506512
H	-0.082960	1.184861	3.359670

Energy: -26.58352205621 Hartrees

$(CH_3)_3P{\cdot}BH_3$

Sy	X	Y	Z
P	0.178940	-0.551215	1.853610
C	1.817630	-1.371053	1.883783
H	2.405596	-1.144869	0.995497
H	2.366500	-1.046244	2.765421
H	1.680079	-2.448150	1.954075
C	-0.556189	-1.083039	0.261400
H	-1.508121	-0.576035	0.117204
H	0.094690	-0.864626	-0.583917
H	-0.749458	-2.153386	0.293795
C	0.571436	1.219861	1.593648
H	1.090823	1.605767	2.468471
H	1.192666	1.377123	0.713159
H	-0.354052	1.780800	1.480318
B	-0.914581	-0.905042	3.390386
H	-0.285102	-0.497337	4.329241
H	-1.933692	-0.296823	3.202020
H	-1.068465	-2.096733	3.403389

Energy: -487.6303648475 Hartrees

$P_3P{\cdot}BH_3$

Sy	X	Y	Z
P	-1.947940	-0.457107	-0.026697
P	-0.003668	0.012567	0.953036
C	-1.360026	-0.587089	-1.819014
H	-1.381621	-1.649149	-2.068335
H	-2.151199	-0.127032	-2.414075
C	-0.000734	-0.003647	-2.228475
H	0.000451	-0.009254	-3.325534
H	-2.360934	0.908850	-0.062903
P	1.371573	-1.445461	-0.018471
C	1.185180	-0.885900	-1.814829
H	2.115273	-0.375920	-2.070693
H	1.180545	-1.806000	-2.402363
C	0.171164	1.467968	-1.827799
P	0.568973	1.924009	-0.036644
H	1.958149	1.596505	-0.063421
H	-0.736769	2.015786	-2.084990
H	0.967950	1.919554	-2.421933
B	-0.006363	0.022804	2.906586
H	-0.869036	0.804800	3.187970
H	1.100210	0.380834	3.193189
H	-0.251704	-1.112608	3.199022
H	0.394225	-2.485710	-0.043025

Energy: -1550.226763454 Hartrees

$P_4{\cdot}BH_3$

Sy	X	Y	Z
P	0.000203	-0.000002	-1.335208
P	0.647614	1.120424	0.418406
P	0.647570	-1.120448	0.418410
P	-1.293040	0.000025	0.418980
B	-0.001309	-0.000000	-3.421010
H	-0.588309	-1.016461	-3.619894
H	1.172228	0.000029	-3.621395
H	-0.588358	1.016433	-3.619889

Energy: -1391.868801377 Hartrees

$P_n{\cdot}0.031(BH_3)$

a	b	c	alpha	beta	gamma
13.4151	9.4621	1.0000	90.00	90.00	90.00

Sy	X	Y	Z
P	0.125019	0.051063	1.080442
P	-0.004078	0.200968	1.022975
P	-0.000062	0.284980	-1.119178
P	0.125019	0.442914	-1.115079

P	-0.125873	0.037571	1.036605
P	0.254117	0.200968	1.022968
P	0.250101	0.284980	-1.119184
P	-0.125480	0.442406	-1.130338
P	0.125019	-0.467399	1.009189
P	-0.001015	-0.311338	1.042600
P	0.000352	-0.213282	-1.044939
P	0.125019	-0.056931	-0.968487
P	-0.126031	-0.467877	0.994281
P	0.251054	-0.311338	1.042599
P	0.249687	-0.213281	-1.044940
P	-0.124653	-0.056016	-1.075758
P	-0.374981	0.036080	1.012943
P	-0.499034	0.194486	1.005676
P	-0.499628	0.284563	-1.123896
P	-0.374981	0.442582	-1.128360
P	0.375911	0.037571	1.036602
P	-0.250927	0.194486	1.005676
P	-0.250333	0.284563	-1.123895
P	0.375519	0.442406	-1.130339
P	-0.374981	-0.465680	0.993943
P	0.499948	-0.308714	1.009561
P	0.499575	-0.213960	-1.097522
P	-0.374981	-0.056985	-1.100582
P	0.376070	-0.467877	0.994280
P	-0.249909	-0.308714	1.009563
P	-0.249536	-0.213959	-1.097520
P	0.374692	-0.056016	-1.075761
B	0.125020	-0.021478	2.965338
H	0.125024	0.091889	3.505473
H	0.200333	-0.086722	3.081567
H	0.049702	-0.086711	3.081598

Energy: -10948.94220513 Hartrees

BAND GAP: 2.32 eV

$P_n \cdot 0.062(BH_3)$

a	b	c	alpha	beta	gamma
6.7089	9.5208	1.0000	90.00	90.00	90.18

Sy	X	Y	Z
P	-0.123712	-0.221765	1.036388
P	-0.379079	-0.071425	0.991614
P	-0.372426	0.011211	-1.159309
P	-0.122594	0.169016	-1.162512
P	0.375947	-0.233151	1.034017
P	0.132942	-0.071369	0.983920
P	0.126405	0.011552	-1.165517
P	0.377919	0.168245	-1.184071
P	-0.122356	0.258764	0.964976
P	-0.373980	0.413547	1.017487
P	-0.373411	-0.485742	-1.059724
P	-0.122803	-0.330569	-1.003321
P	0.377720	0.255950	0.943418
P	0.130825	0.413103	1.012138

P	0.127167	-0.485659	-1.062797
P	0.377454	-0.328758	-1.070382
B	-0.132747	-0.298792	2.900041
H	-0.098826	-0.191378	3.484639
H	-0.005055	-0.384700	2.966679
H	-0.299389	-0.341080	3.007918

Energy: -5487.773073186 Hartrees

BAND GAP: 2.42 eV

P_n·0.125(BH_3)

a	b	c	alpha	beta	gamma
6.7206	9.6149	1.0000	90.00	90.00	90.00

Sy	X	Y	Z
P	-0.835678	-2.054964	1.064308
P	-2.542384	-0.611479	1.050036
P	-2.511315	0.270823	-1.058208
P	-0.835640	1.773834	-1.060383
P	2.524650	-2.136575	1.048883
P	0.870995	-0.611440	1.050186
P	0.840006	0.270765	-1.058122
P	2.524605	1.750469	-0.994579
P	-0.835659	2.672471	1.050896
P	-2.490275	4.196750	1.049628
P	-2.519774	-4.536531	-1.058926
P	-0.835921	-3.056154	-0.993543
P	2.524626	2.752246	1.062948
P	0.818942	4.196808	1.049780
P	0.847797	-4.536579	-1.058811
P	2.524306	-3.034270	-1.062692
B	-0.833158	-2.738025	2.962803
H	-0.838272	-1.652292	3.472871
H	0.182714	-3.345812	3.068649
H	-1.842338	-3.356340	3.069806
B	2.524150	2.070203	2.962098
H	2.525259	3.156437	3.471199
H	-3.184598	1.456225	3.069193
H	1.510830	1.458513	3.068981

Energy: -5514.372894479 Hartrees

BAND GAP: 2.61 eV

P_n·0.250(BH_3)

a	b	c	alpha	beta	gamma
6.8391	9.8829	1.0000	90.00	90.00	89.90

Sy	X	Y	Z
P	-0.154067	-0.211501	1.040775
P	-0.407176	-0.062249	1.074015
P	-0.402981	0.031487	-1.013518
P	-0.153768	0.182744	-0.988013
P	0.347284	-0.213121	1.083462

P	0.097223	-0.063893	1.080555
P	0.096582	0.031744	-0.999520
P	0.346609	0.181670	-0.952972
P	-0.154073	0.288633	1.041952
P	-0.407147	0.437971	1.073081
P	-0.402983	-0.468481	-1.015116
P	-0.153732	-0.317268	-0.989797
P	0.347347	0.287126	1.083574
P	0.097237	0.436246	1.079431
P	0.096568	-0.468304	-1.001488
P	0.346569	-0.318314	-0.954604
B	0.384293	-0.265803	3.120451
H	0.261995	-0.192118	3.550997
H	-0.453162	-0.225961	3.229974
H	0.358745	-0.384237	3.186469
B	0.384136	0.234097	3.115979
H	0.261467	0.307155	3.551626
H	-0.453562	0.274383	3.229605
H	0.359567	0.115531	3.177769
B	-0.112409	-0.308645	2.755828
H	-0.122618	-0.217915	3.539241
H	0.049309	-0.346455	2.529970
H	-0.230018	-0.396042	2.826516
B	-0.112824	0.191369	2.756518
H	-0.122884	0.282002	3.541022
H	0.048771	0.153271	2.531396
H	-0.230798	0.104177	2.825118

Energy: -5567.536066984 Hartrees

BAND GAP: 2.70 eV

I_2

Sy	X	Y	Z
I	0.202162	0.026931	-4.488318
I	-0.196402	-0.029569	-7.335169

Energy: -22.78406057892 Hartrees

$(CH_3)_3P{\cdot}I_2$

Sy	X	Y	Z
I	-3.115248	-1.180700	6.566641
I	-1.374269	-0.800576	4.060824
P	0.198329	-0.456590	1.797345
C	1.799721	-1.345419	1.873835
H	2.383172	-1.181324	0.967918
H	2.368590	-0.997666	2.733102
H	1.619448	-2.411150	1.994599
C	-0.597454	-1.042685	0.253492
H	-1.529502	-0.504423	0.097081
H	0.053953	-0.888239	-0.606684
H	-0.827684	-2.102006	0.341745
C	0.640328	1.292083	1.470125

H	1.185007	1.695464	2.320762
H	1.256964	1.380171	0.575600
H	-0.266254	1.878361	1.338314

Energy: -483.8076624415 Hartrees

$P_3P{\cdot}I_2$

Sy	X	Y	Z
I	-0.000581	0.000339	2.884035
I	-0.007941	-0.000917	5.914146
P	-0.537402	1.933003	-0.951035
P	0.002877	0.000644	0.028412
C	-0.625634	1.341823	-2.745718
H	-1.684883	1.335713	-3.006798
H	-0.178423	2.147508	-3.331239
C	-0.000590	-0.000114	-3.150539
H	-0.001642	-0.000279	-4.247205
H	0.814246	2.390933	-0.965881
P	-1.400673	-1.434215	-0.949160
C	-0.849769	-1.212485	-2.745225
H	-0.316259	-2.126732	-3.009250
H	-1.772787	-1.226364	-3.328172
C	1.474614	-0.129228	-2.747603
P	1.945447	-0.497614	-0.952909
H	1.668406	-1.897718	-0.964405
H	1.998445	0.790812	-3.010846
H	1.947929	-0.920279	-3.332545
H	-2.475380	-0.495028	-0.959763

Energy: -1546.411744208 Hartrees

$P_4{\cdot}I_2$

Sy	X	Y	Z
P	0.083320	0.008820	-1.239060
P	0.627615	1.112631	0.583469
P	0.628513	-1.114803	0.571038
P	-1.295957	-0.001468	0.474485
I	0.019566	0.002169	-4.430706
I	-0.063046	-0.007347	-7.349236

Energy: -1388.062313348 Hartrees

$P_n{\cdot}I_2$

a	b	c	alpha	beta	gamma
6.7089	9.4285	1.0000	90.00	90.00	90.00

Sy	X	Y	Z
P	-0.124845	-0.239096	0.964579
P	-0.377395	-0.083592	0.974488
P	-0.374679	0.005364	-1.158897
P	-0.124839	0.163537	-1.157893
P	0.375158	-0.244129	0.978972
P	0.127706	-0.083598	0.974430

P	0.125001	0.005349	-1.159035
P	0.375147	0.163305	-1.163514
P	-0.124829	0.255523	0.960852
P	-0.375347	0.412995	0.959545
P	-0.375586	-0.494069	-1.154601
P	-0.124839	-0.337361	-1.131884
P	0.375161	0.254909	0.957595
P	0.125673	0.413004	0.959695
P	0.125909	-0.494067	-1.154455
P	0.375159	-0.335134	-1.148575
I	-0.125711	-0.318607	4.023800
I	-0.126945	-0.365034	6.924600

Energy: -5483.960609637 Hartrees

$(CH_3)_3P{\cdot}2I_2$

Sy	X	Y	Z
I	-2.874653	-1.277010	6.734855
I	-1.017200	-0.945189	4.121240
P	0.201431	-0.480462	1.883969
C	1.812022	-1.324087	1.731433
H	2.271401	-1.091693	0.770938
H	2.471133	-1.000110	2.533333
H	1.669554	-2.399101	1.811544
C	-0.829410	-1.016970	0.479571
H	-1.790225	-0.509362	0.522823
H	-0.336981	-0.782229	-0.463661
H	-1.001054	-2.088984	0.541120
C	0.501308	1.305951	1.678837
H	1.145559	1.662707	2.478933
H	0.974703	1.502909	0.717374
H	-0.445222	1.838803	1.732102
I	-4.934266	0.571235	4.935708
I	-6.607501	2.254892	3.089180

Energy: -506.6112171719 Hartrees

$P_3P{\cdot}2I_2$

Sy	X	Y	Z
I	0.014373	-0.348907	2.709528
I	0.181174	-0.320788	5.838992
P	-0.396257	1.851351	-0.733377
P	-0.015602	-0.206133	0.029232
C	-0.511039	1.411846	-2.571478
H	-1.565868	1.500808	-2.833945
H	-0.005109	2.231667	-3.084867
C	0.026224	0.068554	-3.085964
H	0.040949	0.163968	-4.177908
H	0.988308	2.192001	-0.698452
P	-1.528064	-1.444944	-1.039359
C	-0.911020	-1.113725	-2.798751
H	-0.434054	-2.038336	-3.125793
H	-1.819288	-1.017747	-3.396654
C	1.484556	-0.197582	-2.685450

P	1.906924	-0.784569	-0.936395
H	1.496144	-2.143346	-1.085461
H	2.072009	0.707162	-2.846143
H	1.915526	-0.954822	-3.343102
H	-2.511199	-0.412583	-0.980648
I	2.081961	2.419019	5.695879
I	3.777053	4.858405	5.424613

Energy: -1569.211257874 Hartrees

$P_4 \cdot 2I_2$

Sy	X	Y	Z
P	-0.177201	0.050723	-1.057644
P	0.234585	1.186397	0.773515
P	0.621369	-1.010816	0.687999
P	-1.475732	-0.246842	0.685419
I	-0.111829	0.080525	-4.108214
I	-0.034824	0.092385	-7.057597
I	3.391200	0.009980	-7.767116
I	6.221932	-0.056351	-8.499862

Energy: -1410.852122315 Hartrees

$P_n \cdot 2I_2$

a	b	c	alpha	beta	gamma	
6.7091	9.4217	1.0000	90.00	90.00	90.00	

Sy	X	Y	Z
P	-0.126968	-0.234627	0.846713
P	-0.379842	-0.079299	0.856504
P	-0.376753	0.009005	-1.279673
P	-0.126951	0.167335	-1.279940
P	0.373030	-0.240275	0.860838
P	0.125907	-0.079295	0.856593
P	0.122845	0.009003	-1.279582
P	0.373047	0.167053	-1.285557
P	-0.126947	0.259239	0.839038
P	-0.377580	0.416737	0.837515
P	-0.377782	-0.490168	-1.276104
P	-0.126954	-0.333533	-1.248275
P	0.373059	0.258479	0.836041
P	0.123693	0.416727	0.837499
P	0.123872	-0.490172	-1.276094
P	0.373041	-0.331007	-1.268670
I	-0.126661	-0.321103	3.841289
I	-0.126263	-0.389174	6.723944
I	-0.127487	-0.041601	8.099701
I	-0.127406	0.237677	9.361917

Energy: -5506.748987016 Hartrees

η^1 functionalization

$(CO)_5Mo(\eta^1\text{-}P_n)$

a	b	c	alpha	beta	gamma	
13.4124	9.4413	1.0000	90.00	90.00	90.04	

Sy	X	Y	Z
P	0.140381	0.090604	0.794361
P	0.018267	0.253698	0.833113
P	0.014373	0.337518	-1.317623
P	0.140119	0.494993	-1.323608
P	-0.109939	0.101476	1.038814
P	0.265848	0.247031	0.797789
P	0.265552	0.338054	-1.328559
P	-0.109998	0.496536	-1.341714
P	0.141468	-0.414325	0.799045
P	0.016587	-0.257933	0.801088
P	0.013558	-0.158583	-1.270292
P	0.139574	-0.002045	-1.315134
P	-0.109439	-0.414461	0.784606
P	0.264921	-0.254078	0.800299
P	0.264330	-0.160914	-1.314881
P	-0.110069	-0.002122	-1.058919
P	-0.361196	0.091213	0.781106
P	-0.486202	0.247629	0.794380
P	-0.485317	0.339181	-1.327256
P	-0.359682	0.496119	-1.310035
P	0.389943	0.088286	0.799978
P	-0.238713	0.252600	0.811576
P	-0.234601	0.337726	-1.331656
P	0.389902	0.497226	-1.332326
P	-0.361200	-0.414607	0.821995
P	-0.484704	-0.254269	0.809376
P	-0.484410	-0.161938	-1.309431
P	-0.359397	-0.003447	-1.316347
P	0.389950	-0.411365	0.791624
P	-0.235571	-0.258267	0.854428
P	-0.233478	-0.159473	-1.225000
P	0.390290	-0.004144	-1.317826
Mo	-0.112076	0.053410	3.642499
C	-0.141209	0.266353	3.857692
C	0.037025	0.092293	3.908036
C	-0.260946	0.003801	3.771827
C	-0.116639	0.050781	5.623334
O	-0.341097	-0.028517	4.035175
O	0.118124	0.111701	4.230032
O	-0.119577	0.049999	6.774949
O	-0.156202	0.382632	4.119657
C	-0.081607	-0.158352	3.760117
O	-0.064743	-0.275420	3.975215

Energy: -11556.42492145 Hartrees
BAND GAP: 2.24 eV

$Cl_2(CO)Pt(\eta^1-P_n)$

a	b	c	alpha	beta	gamma
13.4232	9.4340	1.0000	90.00	90.00	90.07

Sy	X	Y	Z
P	0.124946	0.048390	1.177129
P	-0.004600	0.198613	1.018324
P	0.001501	0.279683	-1.138964
P	0.126881	0.436927	-1.159835

P	-0.125899	0.034621	0.958307
P	0.257068	0.197096	1.030975
P	0.249833	0.275803	-1.151394
P	-0.124386	0.436778	-1.167305
P	0.126297	-0.472747	0.962816
P	0.000015	-0.318319	0.952760
P	0.002067	-0.216262	-1.108023
P	0.126204	-0.060489	-0.895292
P	-0.126044	-0.472717	0.951606
P	0.252054	-0.315228	1.027710
P	0.249704	-0.216967	-1.062902
P	-0.123689	-0.059465	-1.148556
P	-0.374275	0.029706	0.957554
P	-0.498129	0.188108	0.963724
P	-0.499219	0.280114	-1.158888
P	-0.374654	0.439057	-1.180532
P	0.377680	0.029233	0.961447
P	-0.251258	0.190037	0.951654
P	-0.250284	0.280042	-1.177310
P	0.374650	0.435726	-1.144778
P	-0.374423	-0.469150	0.941553
P	-0.499501	-0.311994	0.957785
P	0.499829	-0.219277	-1.164284
P	-0.374343	-0.061952	-1.163676
P	0.377084	-0.472613	0.984832
P	-0.249089	-0.311919	0.953754
P	-0.248448	-0.218678	-1.159721
P	0.375281	-0.059464	-1.169357
Pt	0.118301	-0.032085	3.455163
Cl	0.118063	-0.094905	5.762629
Cl	0.276175	0.074127	3.835840
C	-0.010040	-0.114569	3.572339
O	-0.085053	-0.163062	3.843220

Energy: -12075.33947754 Hartrees
BAND GAP: 2.20 eV

$(CO)_4Ru(\eta^1\text{-}P_n)$

a	b	c	alpha	beta	gamma
13.4128	9.4579	1.0000	90.00	90.00	90.00

Sy	X	Y	Z
P	0.140331	0.091250	0.863986
P	0.018606	0.254384	0.864737
P	0.014387	0.335698	-1.296001
P	0.139598	0.493988	-1.292354
P	-0.110551	0.103989	1.073271
P	0.265673	0.247329	0.840300
P	0.265016	0.337202	-1.290516
P	-0.110594	0.493764	-1.313664
P	0.140597	-0.415733	0.833573
P	0.014950	-0.260611	0.847265
P	0.013214	-0.158845	-1.215638
P	0.139109	-0.002535	-1.245653
P	-0.110563	-0.416519	0.806794
P	0.264135	-0.255718	0.844127
P	0.263710	-0.161275	-1.264151

P	-0.110583	-0.002824	-1.010684
P	-0.361491	0.091201	0.864393
P	-0.486848	0.247247	0.841619
P	-0.486190	0.337153	-1.289413
P	-0.360786	0.493941	-1.291434
P	0.389386	0.088232	0.848865
P	-0.239756	0.254364	0.865685
P	-0.235547	0.335690	-1.295169
P	0.389427	0.495708	-1.294859
P	-0.361753	-0.415776	0.834175
P	-0.485294	-0.255761	0.844660
P	-0.484846	-0.161291	-1.263572
P	-0.360264	-0.002552	-1.245113
P	0.389413	-0.412575	0.828117
P	-0.236137	-0.260612	0.847571
P	-0.234359	-0.158859	-1.215550
P	0.389434	-0.004469	-1.266894
Ru	-0.110373	0.056910	3.592528
C	-0.110611	0.265418	3.788311
C	0.015902	-0.051772	3.644549
C	-0.236590	-0.051892	3.647198
C	-0.110096	0.049809	5.503235
O	-0.307904	-0.113912	3.891378
O	0.087235	-0.113745	3.889135
O	-0.109878	0.046998	6.647432
O	-0.110706	0.383801	4.044064

Energy: -11469.47004206 Hartrees
BAND GAP: 2.22 eV

ClAu(η^1-P_n)

a	b	c	alpha	beta	gamma
6.7097	9.4863	1.0000	90.00	90.00	90.00

Sy	X	Y	Z
P	-0.125047	-0.211636	1.023115
P	-0.379662	-0.060024	1.103195
P	-0.373271	0.026654	-1.039439
P	-0.125029	0.185552	-0.996453
P	0.374970	-0.221397	1.146581
P	0.129596	-0.060038	1.103435
P	0.123195	0.026651	-1.039258
P	0.374968	0.182683	-1.086658
P	-0.125020	0.275009	1.143044
P	-0.378205	0.430389	1.143182
P	-0.375535	-0.473729	-0.964472
P	-0.125026	-0.317365	-1.032774
P	0.374975	0.270938	1.029650
P	0.128179	0.430389	1.143002
P	0.125477	-0.473729	-0.964642
P	0.374975	-0.316248	-0.962886
Au	-0.125114	-0.363922	2.740424
Cl	-0.124431	0.484426	4.567052

Energy: -6056.724780848 Hartrees

BAND GAP: 2.17 eV

ClAu(Me_2S)

Sy	X	Y	Z
Au	-0.413990	0.050575	2.455670
Cl	-1.669732	-0.143078	4.383051
S	0.825705	0.253113	0.571225
C	-0.272268	-0.248782	-0.817281
H	0.313201	-0.292366	-1.731782
H	-0.736589	-1.205715	-0.603909
H	-1.037296	0.516296	-0.902552
C	1.959998	-1.195539	0.555947
H	2.492418	-1.217037	-0.391168
H	2.660424	-1.051924	1.372411
H	1.402029	-2.112643	0.712187

Energy: -1073.348460694 Hartrees

Me_2S

Sy	X	Y	Z
S	0.007429	0.079122	0.001573
C	1.787953	-0.087747	-0.276636
H	1.947667	-0.059194	-1.353989
H	2.334090	0.736614	0.183290
H	2.156203	-1.038734	0.109953
C	0.021244	0.003453	1.809805
H	0.600753	0.825910	2.230608
H	-1.012388	0.094089	2.141942
H	0.421856	-0.949310	2.157830

Energy: -476.9610484976 Hartrees

ClAu(Me_3P)

Sy	X	Y	Z
Au	-0.000007	-0.000074	3.073370
Cl	-0.000136	-0.000059	5.397772
P	0.000037	-0.000067	0.846510
C	1.654928	0.051506	0.052523
C	-0.872015	1.407382	0.052560
C	-0.782881	-1.458958	0.052534
H	1.578235	0.049061	-1.034347
H	2.241366	-0.809302	0.367033
H	2.186434	0.947301	0.366934
H	-0.832004	1.341927	-1.034310
H	-0.419312	2.345557	0.366672
H	-1.913411	1.420218	0.367388
H	-0.746657	-1.391331	-1.034339
H	-1.821576	-1.536188	0.367032
H	-0.273099	-2.367272	0.366967

Energy: -1056.560538467 Hartrees

η^2 functionalization

$(CH_3)_2Ni(H_2O)_2$

Sy	X	Y	Z
Ni	1.065541	-1.251107	2.574141

C	0.205445	-2.348666	3.882798
H	0.782975	-2.478937	4.798134
H	-0.030693	-3.380217	3.551476
H	-0.758805	-1.925114	4.180955
C	1.643294	-0.068608	3.956125
H	2.057184	0.893333	3.593143
H	2.441240	-0.512112	4.556544
H	0.848696	0.211368	4.649791
O	0.448041	-2.584401	1.087307
H	-0.405205	-2.343020	0.703838
H	0.254734	-3.258276	1.760191
O	2.034499	-0.070104	1.190703
H	2.245890	0.678243	1.768287
H	1.585564	0.265917	0.406666

Energy: -1740.841585261 Hartrees

H_2O

Sy	X	Y	Z
O	-0.002857	-0.094744	0.000000
H	0.953839	-0.006054	0.000000
H	-0.350982	0.800798	0.000000

Energy: -76.18811196351 Hartrees

$(CH_3)_2Ni(\eta^2\text{-}P_n)$

a	b	c	alpha	beta	gamma
6.7068	9.2749	1.0000	90.00	90.00	91.06

Sy	X	Y	Z
P	0.268205	0.025550	1.030056
P	0.029230	0.190817	1.172739
P	0.027723	0.286704	-0.926013
P	0.283762	0.444720	-0.926262
P	-0.220161	0.027147	1.137179
P	-0.471854	0.183922	1.218286
P	-0.470440	0.284688	-0.879611
P	-0.218107	0.446750	-0.879651
P	0.282162	-0.459252	1.172189
P	0.043202	-0.294001	1.028207
P	0.029397	-0.214113	-1.147892
P	0.282028	-0.054248	-1.146859
P	-0.216698	-0.452373	1.217926
P	-0.468428	-0.295591	1.136973
P	-0.470317	-0.214880	-1.037966
P	-0.218299	-0.053492	-1.037834
Ni	0.156286	-0.134573	2.536237
C	0.000662	-0.223473	3.963682
H	-0.005593	-0.154865	4.842331
H	0.062598	-0.326298	4.269144
H	-0.152104	-0.243611	3.622862
C	0.313157	-0.047130	3.964836
H	0.464367	-0.023036	3.614843
H	0.323950	-0.118413	4.822185
H	0.248270	0.053350	4.305712

Energy: -7049.186116151 Hartrees

BAND GAP: 2.31 eV

NH_3

Sy	X	Y	Z
N	-0.961247	-0.567464	1.195082
H	-0.903146	-1.553919	1.430037
H	-1.892698	-0.428093	0.814789
H	-0.947008	-0.070825	2.080992

Energy: -56.52158650822 Hartrees

$(CO)_2Ni(NH_3)_2$

Sy	X	Y	Z
Ni	0.302788	0.229675	0.182882
C	1.185086	1.267051	1.260789
O	1.724705	2.097641	1.870742
C	-0.460126	1.017956	-1.163655
O	-0.898605	1.701035	-1.996924
N	1.581604	-1.369896	-0.519315
H	2.089455	-1.909979	0.175236
H	2.283230	-0.851680	-1.038887
H	1.187057	-2.040287	-1.172512
N	-1.156186	-0.926203	1.288842
H	-0.842096	-1.424290	2.116421
H	-1.741170	-1.573524	0.768445
H	-1.775942	-0.193700	1.620338

Energy: -1847.954759163 Hartrees

$(CO)_2Ni(\eta^2\text{-}P_n)$

a	b	c	alpha	beta	gamma
6.7069	9.3906	1.0000	90.00	90.00	92.01

Sy	X	Y	Z
P	0.284988	0.043700	0.946785
P	0.047544	0.208290	0.948106
P	0.048698	0.300087	-1.156658
P	0.305386	0.459149	-1.156385
P	-0.207539	0.048559	0.899163
P	-0.452686	0.203454	1.001136
P	-0.452263	0.300548	-1.107485
P	-0.193555	0.458789	-1.108491
P	0.306495	-0.448967	0.947933
P	0.069118	-0.284300	0.945889
P	0.050640	-0.199680	-1.213520
P	0.303522	-0.040851	-1.213199
P	-0.193147	-0.444081	0.999856
P	-0.438363	-0.289273	0.897471
P	-0.451030	-0.199655	-1.230463
P	-0.194885	-0.040954	-1.229294
Ni	0.177364	-0.120222	2.421260
C	0.366409	-0.184125	3.547495
C	-0.011909	-0.056834	3.548584

O	0.471205	-0.218358	4.382215
O	-0.116491	-0.023375	4.387200

Energy: -7196.086111180 Hartrees

BAND GAP: 2.22 eV

$(CO)_4Mo((CH_3)_2S)_2$

Sy	X	Y	Z
Mo	-0.117730	0.004382	-0.207455
C	-0.283172	-2.025268	-0.287517
C	-2.076062	0.093687	-0.397860
C	-0.069805	0.006772	-2.176536
O	-3.223257	0.143973	-0.564696
O	-0.095013	0.012370	-3.336562
O	-0.442360	-3.165651	-0.383769
C	-0.092888	2.040968	-0.281016
O	-0.122770	3.190658	-0.390812
S	2.458303	-0.148153	-0.233008
C	3.187184	-1.265933	1.028924
H	2.749076	-2.247473	0.871585
H	2.973935	-0.926014	2.039040
H	4.262298	-1.323685	0.878712
C	3.287993	1.407338	0.278187
H	2.992369	2.169781	-0.435770
H	4.365235	1.269624	0.233033
H	2.987108	1.711021	1.276716
S	-0.449306	0.048815	2.351370
C	0.752184	1.086365	3.275097
H	0.681213	2.090182	2.865868
H	1.768801	0.717068	3.167951
H	0.475335	1.105821	4.326212
C	-0.095582	-1.558172	3.165229
H	-0.794622	-2.279099	2.752625
H	-0.265818	-1.462499	4.234465
H	0.920853	-1.888476	2.971785

Energy: -1476.404684100 Hartrees

$(CO)_4Mo(\eta^2\text{-}P_n)$

a	b	c	alpha	beta	gamma
13.3984	9.4275	1.0000	90.00	90.00	88.72

Sy	X	Y	Z
P	0.129091	0.052184	0.835715
P	0.002612	0.208376	0.942653
P	0.001912	0.299482	-1.161578
P	0.120626	0.464002	-1.079176
P	-0.116772	0.042591	1.042396
P	0.250260	0.209104	0.897673
P	0.248818	0.307084	-1.185863
P	-0.128657	0.450732	-1.161551
P	0.123613	-0.446504	1.075634
P	-0.009943	-0.292391	1.041914
P	-0.001201	-0.203525	-1.104786
P	0.122605	-0.046478	-1.242610
P	-0.129325	-0.458168	0.942717

P	0.248187	-0.290193	0.966884
P	0.249860	-0.204653	-1.179757
P	-0.125538	-0.046262	-1.104329
P	-0.374921	0.040423	0.967533
P	0.499022	0.201558	0.953819
P	0.498024	0.291666	-1.171946
P	-0.375537	0.443155	-1.185276
P	0.374479	0.046602	0.930667
P	-0.250331	0.196718	1.076137
P	-0.247364	0.286210	-1.078758
P	0.375263	0.458569	-1.172132
P	-0.376990	-0.458874	0.898358
P	0.498782	-0.296383	0.930925
P	-0.499622	-0.203872	-1.189195
P	-0.376583	-0.045131	-1.179065
P	0.374235	-0.451333	0.953650
P	-0.255807	-0.301953	0.837015
P	-0.249340	-0.203312	-1.241471
P	0.372904	-0.045916	-1.189427
Mo	-0.063293	-0.124912	2.947201
C	-0.092432	0.024849	4.307598
C	0.081327	-0.077456	3.398340
C	-0.207820	-0.172547	3.402028
C	-0.033774	-0.274605	4.307200
O	-0.281472	-0.192657	3.958463
O	0.155096	-0.057667	3.953113
O	-0.018863	-0.362451	5.087076
O	-0.106931	0.112839	5.087311

Energy: -11443.10688342 Hartrees

η[3]functionalization

(CO)$_3$Mo(η^6-p-xylene)

Sy	X	Y	Z
Mo	2.383210	3.721898	3.229509
C	3.875617	4.539766	4.197587
C	1.408234	5.418617	3.341515
O	4.764797	4.966649	4.815040
O	0.768584	6.385992	3.425980
C	3.193153	4.323575	1.551595
C	1.987365	2.112388	4.948441
C	1.281929	1.884910	2.176851
C	0.746830	2.554092	4.479637
H	0.036269	2.986736	5.170983
C	2.896607	1.552046	3.999555
H	3.864078	1.205815	4.337921
C	2.553478	1.440608	2.652884
H	3.256475	1.008627	1.953206
C	0.396652	2.441234	3.104673
H	-0.573211	2.790408	2.776881
O	3.660027	4.616614	0.526851
C	2.345030	2.170177	6.402818
H	2.139759	1.212454	6.886907
H	1.771122	2.934442	6.923024
H	3.401598	2.390010	6.544699

C	0.902888	1.707035	0.737687
H	0.506158	0.703538	0.566291
H	1.760823	1.838067	0.080785
H	0.139129	2.421502	0.436982

Energy: -718.1697314172 Hartrees

p-xylene

Sy	X	Y	Z
C	2.051039	2.066456	4.894473
C	1.228314	1.859131	2.211151
C	0.811499	2.538723	4.486777
H	0.151242	3.000404	5.214453
C	2.873685	1.491089	3.934990
H	3.852334	1.121134	4.224627
C	2.470015	1.389328	2.618470
H	3.135889	0.940757	1.887826
C	0.407599	2.436985	3.169385
H	-0.565427	2.819783	2.877287
C	2.480165	2.150990	6.332377
H	2.224408	1.242303	6.883550
H	1.998912	2.981846	6.848112
H	3.558388	2.283540	6.423689
C	0.779258	1.722511	0.783364
H	0.305053	0.754714	0.600730
H	1.615912	1.800901	0.088863
H	0.052214	2.488706	0.514676

Energy: -310.7093311416 Hartrees

$(CO)_3Mo(PMe_3)$

Sy	X	Y	Z
Mo	0.432353	0.001056	-0.006182
C	1.615135	1.579829	-0.054462
C	1.636105	-0.741539	1.369781
C	1.622857	-0.813607	-1.352618
O	2.355784	2.481175	-0.091037
O	2.388383	-1.157739	2.159216
O	2.368106	-1.285161	-2.117143
P	-0.895334	-2.157433	-0.028002
P	-0.924603	1.092330	-1.847690
P	-0.903789	1.044207	1.877888
C	-2.711126	0.704201	-2.155873
H	-3.119350	1.303005	-2.970682
H	-2.832520	-0.345895	-2.417378
H	-3.300513	0.897318	-1.261226
C	-0.244644	0.862331	-3.547307
H	-0.249656	-0.193214	-3.812197
H	-0.815655	1.415972	-4.293086
H	0.789187	1.199832	-3.570433
C	-1.012188	2.937955	-1.782815
H	-1.526217	3.349564	-2.651711
H	-1.538047	3.263435	-0.886821
H	-0.006053	3.349150	-1.741999
C	-0.190045	-3.507332	1.013245
H	-0.192412	-3.211478	2.060539

H	-0.748836	-4.437630	0.908060
H	0.844489	-3.681003	0.725406
C	-2.677582	-2.255789	0.473083
H	-3.072917	-3.265995	0.362152
H	-2.796476	-1.961171	1.514652
H	-3.279884	-1.584158	-0.136226
C	-0.981204	-3.021131	-1.660455
H	-1.478732	-3.988022	-1.581138
H	-1.522841	-2.414803	-2.384541
H	0.024737	-3.173781	-2.045017
C	-0.971579	0.061977	3.442851
H	-1.474514	0.607546	4.241667
H	-1.500655	-0.875796	3.281800
H	0.038677	-0.180584	3.764695
C	-0.216960	2.630289	2.523273
H	-0.231693	3.388822	1.743016
H	-0.777801	2.997570	3.383103
H	0.820530	2.481471	2.814327
C	-2.692743	1.504082	1.718706
H	-3.089630	1.909095	2.650167
H	-2.824368	2.256329	0.942398
H	-3.284975	0.632522	1.445216

Energy: -1790.502757016 Hartrees

$(CO)_3Mo(\eta^3-P_n)$

a	b	c	alpha	beta	gamma
13.3714	9.3750	1.0000	90.00	90.00	90.00

Sy	X	Y	Z
P	0.124971	0.041051	1.031149
P	0.000472	0.203214	1.096704
P	-0.003480	0.291376	-1.041947
P	0.124992	0.443815	-1.229279
P	-0.125298	0.046014	1.068911
P	0.249491	0.203192	1.096765
P	0.253458	0.291371	-1.041786
P	-0.122824	0.459850	-0.944800
P	0.124990	-0.464752	0.861545
P	0.011092	-0.292816	0.988515
P	0.000048	-0.199762	-1.137354
P	0.124989	-0.042273	-1.159898
P	-0.121865	-0.442824	1.183035
P	0.238891	-0.292813	0.988860
P	0.249929	-0.199756	-1.137074
P	-0.126027	-0.041818	-1.065764
Mo	0.124953	-0.159727	2.613836
C	0.124966	-0.322568	3.915526
O	0.125018	-0.411694	4.709429
C	0.230692	-0.072545	3.742990
O	0.289974	-0.023995	4.449756
C	0.019237	-0.072568	3.743543
O	-0.040032	-0.024059	4.450715

Energy: -11329.80941363 Hartrees
BAND GAP: 2.34 eV
